# Global Burden of Oral Disorders in Adults Aged ≥65, 1990 to 2021: A Systematic Analysis of Global Burden of Disease Study 2021

**DOI:** 10.1016/j.identj.2025.109345

**Published:** 2025-12-24

**Authors:** Yilin Zhang, Xin Xu, Weilu Pan, Cheng Lv, Yuan Tang, Jing Li

**Affiliations:** a School of Stomatology, Shandong Second Medical University, Weifang, Shandong, P.R. China; b Affiliated Hospital of Shandong Second Medical University, Weifang, Shandong, P.R. China; c School of Public Health, Shandong Second Medical University, Weifang, Shandong, P.R. China

**Keywords:** Disability-adjusted life-years, Health inequality, Oral disease, Sociodemographic index, Global burden of disease

## Abstract

**Aim:**

This study aimed to examine the global burden, trends, and socioeconomic inequalities associated with oral disorders in adults aged 65 years and older from 1990 to 2021, and to project future trends to inform targeted public health strategies.

**Methods:**

Using data from the Global Burden of Disease Study 2021, we examined age-standardized DALY rates for oral conditions. Analytical methods included Joinpoint regression (for trend analysis), the slope index of inequality and concentration index(for inequality assessment), frontier analysis (for efficiency assessment), and autoregressive integrated moving average modelling (to project trends through 2035).

**Conclusion:**

Absolute case counts for all oral diseases increased significantly (by 112%-142%) from 1990 to 2021. Although age-standardized disability rates declined for edentulism, dental caries, and other oral disorders, they remained stagnant for periodontal diseases and lip/oral cavity cancer. Socioeconomic disparities persisted or even widened, with disease-specific patterns. Projections to 2035 indicate a growing burden among older populations, highlighting an urgent need for equitable and targeted public health interventions.

**Clinical relevance**

The findings highlight the critical importance of context-specific policies: basic preventive and prosthetic care in low-resource settings, and optimized, integrated geriatric oral healthcare in high-resource regions. Clinicians should prioritize early restorative care and preventive strategies, especially for high-risk elderly populations.

Alt-text: Unlabelled box

## Introduction

Oral disorders represent a major global public health challenge, disproportionately affecting older adults.^1^ This population is particularly vulnerable to multiple conditions, including caries of permanent teeth, periodontal diseases, edentulism, and lip and oral cavity cancer.^2^ As global aging accelerates, the burden of these diseases continues to rise.^3^ Their consequences extend beyond impaired mastication, swallowing, and speech, ultimately contributing to malnutrition, psychological distress, and social impairment.^4^ For example, untreated caries and periodontal disease can lead to pain, infections, and dietary restrictions,^5^^,^^6^ whereas edentulism compromises chewing efficiency and may worsen nutritional status.^7^ Oral cancers are associated with increased mortality risks.^8^ Furthermore, oral diseases are linked to systemic conditions such as cardiovascular disease and diabetes, compounding the overall healthcare burden.^9^ High treatment demands place considerable strain on healthcare resources and impose economic costs on families and societies.^10^ Therefore, understanding global epidemiological trends is essential for the development of effective public health strategies.

Recent studies using the Global Burden of Disease Study (GBD) data have significantly advanced oral health research, ranking oral disorders as the 11th leading cause of disability worldwide. In 2021, these conditions accounted for 3.69 billion prevalent cases and 23.2 million years lived with disability^2^ The present study aimed to provide a more integrated perspective specifically tailored for the elderly population. Although existing literature offers valuable insights, there remains a need for better long-term trend analyses, detailed assessments of socioeconomic disparities, and projections of future disease burden in this growing demographic.^11^

This study, therefore, aimed to provide a comprehensive analysis of the global burden, trends, and socioeconomic inequalities associated with oral disorders in adults aged 65 years and older from 1990 to 2021, and to project future trends up to 2035. The findings are expected to strengthen the evidence base needed to develop targeted and equitable public health strategies aiming at improving the oral health among aging populations.

## Methods

### Data source

The study on the global burden of oral diseases in the elderly population within the GBD project is classified as a large-scale descriptive epidemiological study utilizing GBD data. This work constitutes a specific subanalysis of the GBD 2021 dataset, with a dedicated focus on adults aged 65 years and older. The GBD study 2021 provides estimates of the global burden of 371 diseases and injuries across 204 countries and territories between 1990 and 2021. The GBD 2021 integrates data from diverse sources, including scientific literature, vital registration systems, verbal autopsy records, household surveys, and disease registries, to produce estimates for oral disorders. The complete GBD study methodology, encompassing data collection procedures, estimation methods, and model fit evaluation, is thoroughly described in the main report of the GBD 2021 Disease and Injury Collaborative Group and its corresponding appendix.^12^ Data on oral diseases, including caries of permanent teeth, periodontal diseases, edentulism, lip and oral cavity cancer, and other oral disorders, were obtained from the GBD 2021 using the analytical tools on the Global Health Data Exchange platform (GHDx). The study population was defined as adults aged 65 years and older. GBD cases were defined as follows: any tooth with significant coronal caries involving dentin or root caries involving dentin with soft or leathery upon probing (caries of permanent teeth); periodontal probing depth ≥6 mm or loss of attachment ≥6 mm (periodontal diseases); complete loss of permanent teeth (edentulism); ICD-10 codes C00-C08.9, D10.0-D10.5, and D11-D11-9 (lip and oral cavity cancer); and other diseases or malformations of the teeth, tongue, and jaws not captured by the above criteria (other oral disorders). The specific disease modelling method has been described in previous studies.^2^

GBD 2021 draws oral disease data from several major sources, including vital registration systems, verbal autopsies, national censuses, household surveys, disease-specific registries, health service linkage data, and other relevant datasets.^12^ In the present study, people aged 65 years and older were categorized into seven GBD-defined age groups, each spanning 5 years.

### Statistical analysis

We utilized a comprehensive suite of statistical models to analyse the trends, inequalities, and future projections of the oral disease burden. The temporal trend of age-standardized disability rate (ASDR) was assessed by calculating the estimated annual percentage change (EAPC) across the entire study period from 1990 to 2021.^13^ To further identify specific inflection points in these temporal trends, we performed a Joinpoint regression analysis. This method fits a series of connected straight-line segments to the data on a log-linear scale, identifying the years in which significantly changes in the trend occur. The optimal number of segments, ranging from 0 to 5, was determined using a Monte Carlo permutation test. For each segment, we present the APC, and the overall trend across the study period is summarized by the average annual percentage change (AAPC) along with its 95% confidence interval (CI).^14^ This Joinpoint analysis was performed not only at the global level but also separately for each of the five sociodemographic index (SDI) regions, in order to identify divergent patterns across different developmental strata.^15^

To assess health inequalities, we utilized two well-established indices. The slope index of inequality (SII) was calculated to quantify the absolute inequality across countries. This index was derived by performing a robust regression of each country’s disability-adjusted life year (DALY) rate against its relative position in the global SDI ranking, which scaled from 0 (lowest) to 1 (highest). The resulting SII reflects the absolute disparity in DALY rates between the hypothetical most and least disadvantaged countries. At the same time, we assessed the relative inequality across the global population using the concentration index (CIx). The CIx is defined as twice the area between the concentration curve – which plots the cumulative proportion of DALYs against the cumulative proportion of the population ranked by SDI – and the line of perfect equality. A negative CIx value indicates that the disease burden is disproportionately concentrated among populations with lower socioeconomic status.^16^

Furthermore, we performed a frontier analysis to assess the potential for reducing the disease burden in relation to each region’s socioeconomic development level. This approach estimates the theoretical minimum achievable disease burden at each SDI level by benchmarking against the world’s best-performing countries and regions. The resulting frontier provides as a reference for assessing the performance of other nations and quantifying potential health improvements. Technically, the frontier was constructed using a combination of locally estimated scatterplot smoothing (LOESS) and local polynomial regression, with smoothing spans ranging from 0.3 to 0.5. This curve represents the lowest achievable ASDR for a given SDI, as defined by the best-performing countries. For each country in 2021, we calculated the gap between its observed ASDR and the corresponding theoretical minimum. The uncertainty associated with the gap estimates was assessed using bootstrap methods. Countries with the largest gaps were identified as having the greatest potential for health improvement.^17^

Finally, autoregressive integrated moving average (ARIMA) models were used to forecast the future disease burden through 2035. These models incorporated both autoregressive and moving average components, with the best-fitting model for each age group and disease selected based on standard model selection criteria, such as the Akaike information criterion. All data processing, statistical analyses, and visualizations were performed using R software (version 4.5.1), and statistical uncertainties were rigorously validated through posterior distribution sampling and Monte Carlo methods in accordance with the GBD analytical framework.^18^

## Result

### Oral diseases

**Fig. 1:**
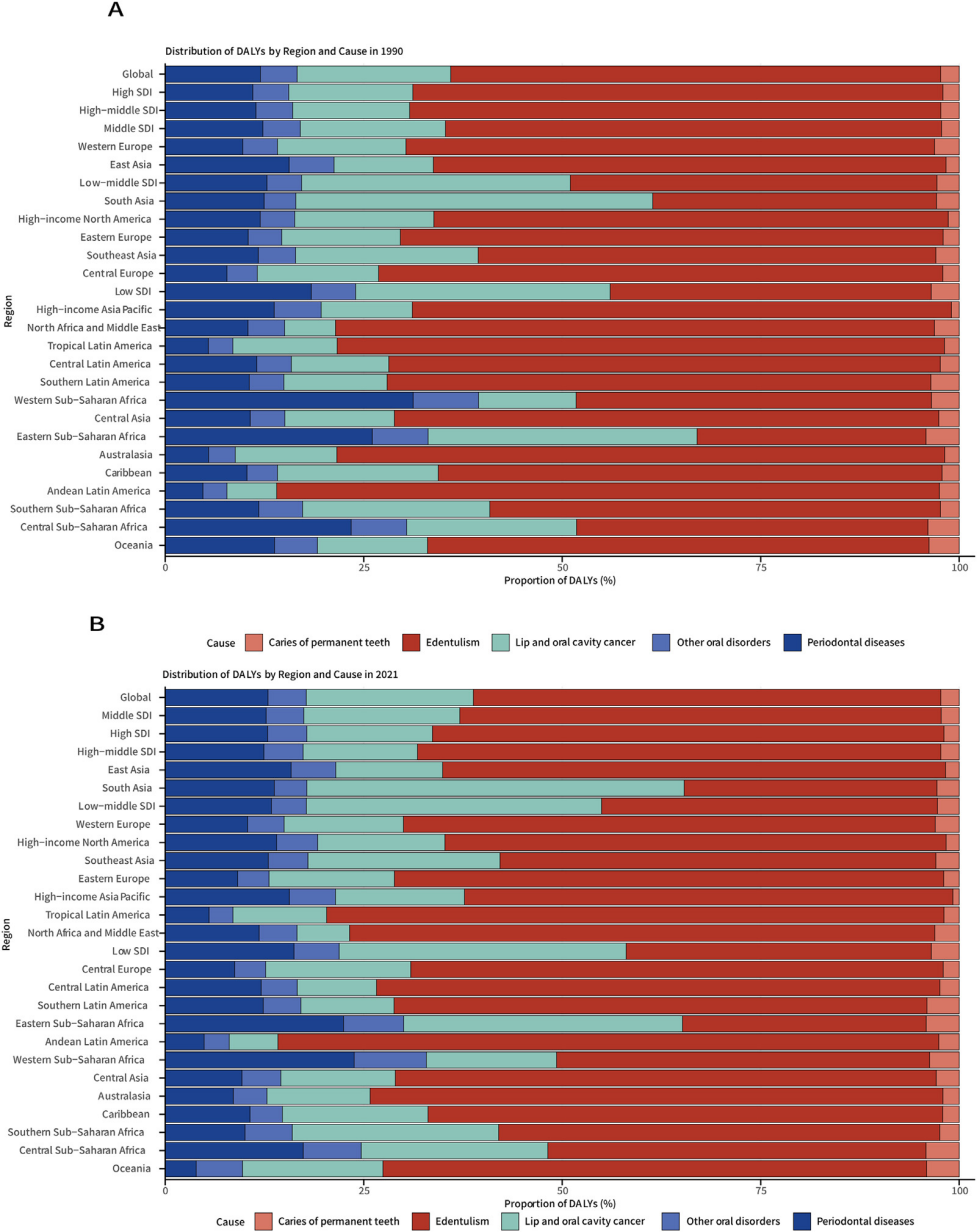
Distribution of DALYs attributable to oral disorders in older adults across 21 GBD regions and five SDI quintiles, 1990（A) and 2021(B). DALYs, disability-adjusted life-years; SDI, sociodemographic index.

**Fig. 2:**
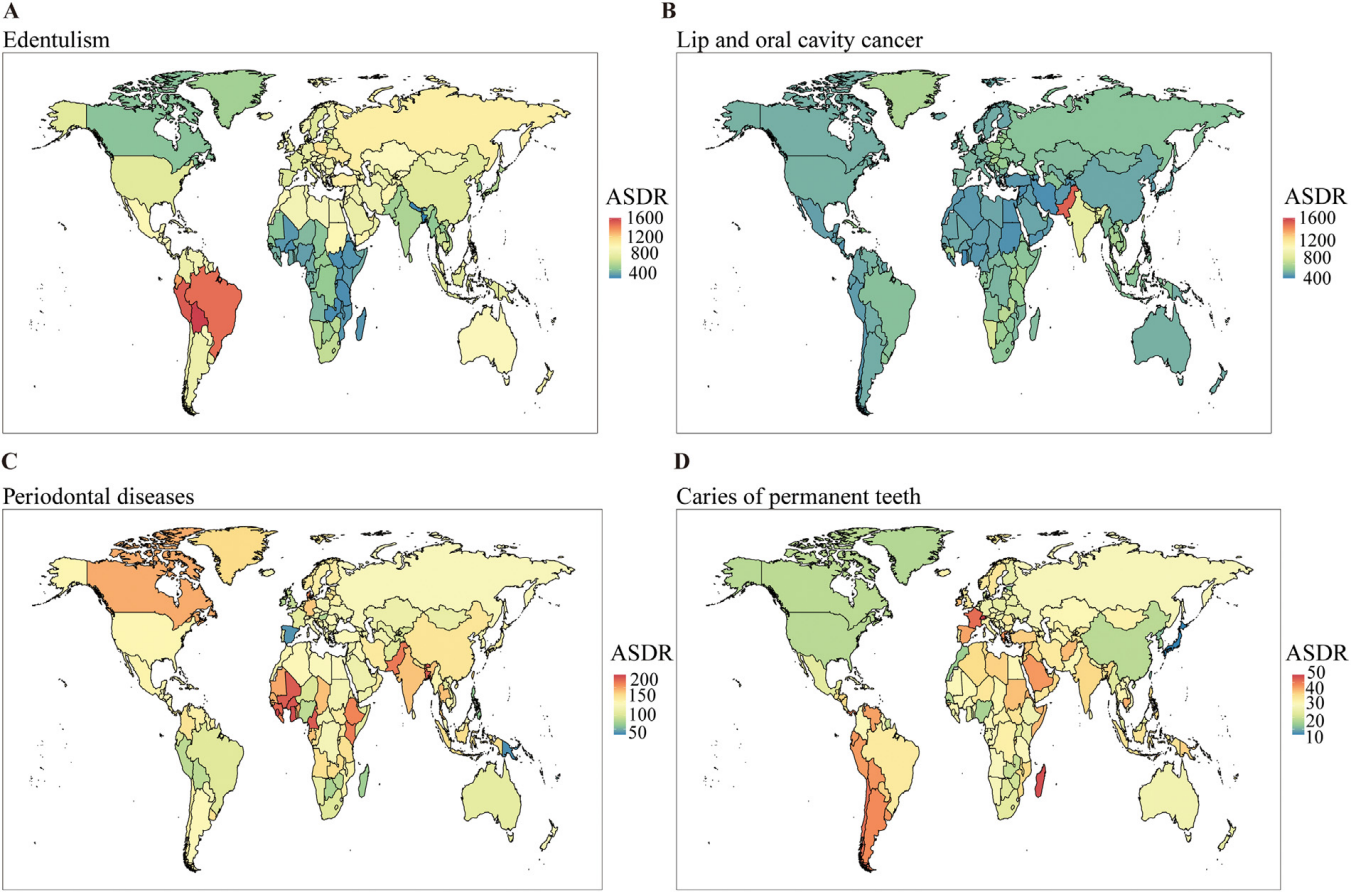
Global distribution of ASDR for oral disorders in 2021. (A) Edentulism; (B) Lip and oral cavity cancer; (C) Periodontal diseases; (D) Caries of permanent teeth. Note: ASDR, age-standardized disability-adjusted life years (DALYs) rate.

Between 1990 and 2021, the global older adult population exhibited substantial increases in absolute case numbers for all major oral diseases: periodontal diseases (+140%; 497,436-1193,527 cases), edentulism (+112%; 2558,666-5429,544 cases), lip and oral cavity cancer (+142%; 801,473-1940,092 cases), caries of permanent teeth (+121%; 96,617-213,670 cases), and other oral disorders (+132%; 191,413-444,114 cases). Analysis of ASDR revealed distinct trends: significant declines were observed in edentulism (EAPC = –0.47, 95% CI: –0.58 to –0.36), caries of permanent teeth (EAPC = –0.12, 95% CI: –0.18 to –0.06), and other oral disorders (EAPC = –0.02, 95% CI: –0.03 to –0.02), whereas periodontal diseases (EAPC = 0.07, 95% CI: –0.02 to 0.17) and lip and oral cavity cancer (EAPC = 0.03, 95% CI: –0.02 to 0.08) remained stable. In 2021, edentulism continued to exhibit the highest ASDR burden, followed by lip and oral cavity cancer, periodontal diseases, other oral disorders, and caries of permanent teeth (Fig. 1, Fig. 2 and Fig. 1, Fig. 2, Supplementary Figure 1 and Supplementary Table 1).

### Edentulism

**Fig. 3:**
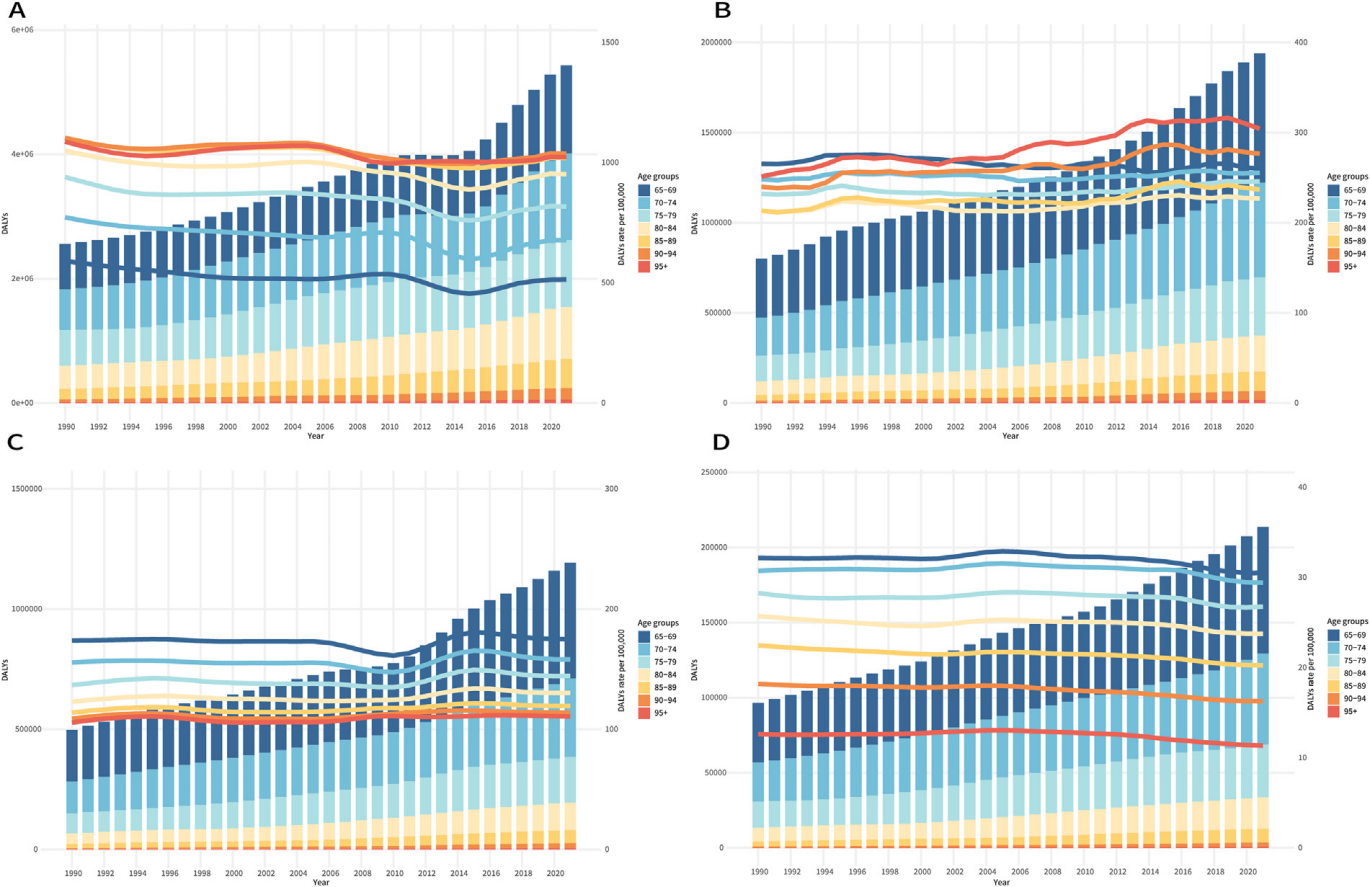
The number of DALYs and the ASDR for the global burden of oral disorders across different age groups (65-69, 70-74, 75-79, 80-84, 85-89, 90-94, and ≥95 years) from 1990 to 2021. (A) Edentulism, (B) lip and oral cavity cancer, (C) periodontal diseases, and (D) caries of permanent teeth. ASDR, age-standardized DALYs rate; DALYs, disability-adjusted life years.

From 1990 to 2021, the global DALY cases due to edentulism increased by 112.2% (25.6-54.3 million), while the ASDR decreased significantly (EAPC = –0.47, 95% CI: –0.58 to –0.36). Gender disparities remained evident: although females continued to experience a higher burden (2021 ASDR: 756.53/100,000 vs 643.21/100,000 in males), males showed a faster decline (EAPC: –0.49 vs –0.44). SDI analysis revealed the steepest reductions in high-SDI regions (EAPC = –0.93, 95% CI: –1.22 to –0.63), whereas low-SDI regions exhibited minimal change (EAPC = –0.08, 95% CI: –0.50 to 0.35). Geographically, Andean Latin America recorded the highest ASDR in 2021 (1503.56/100,000), while Eastern Europe was the only region showing an increasing burden (EAPC = 0.14, 95% CI: 0.07-0.22) (Supplementary Table 2). Among elderly age groups, the 65 to 69 years group accounted for the highest number of DALY cases, whereas the 90 to 94 years group exhibited the highest ASDR. Fluctuation in ASDR across age groups were observed in different years (Figure 3A).

**Fig. 4:**
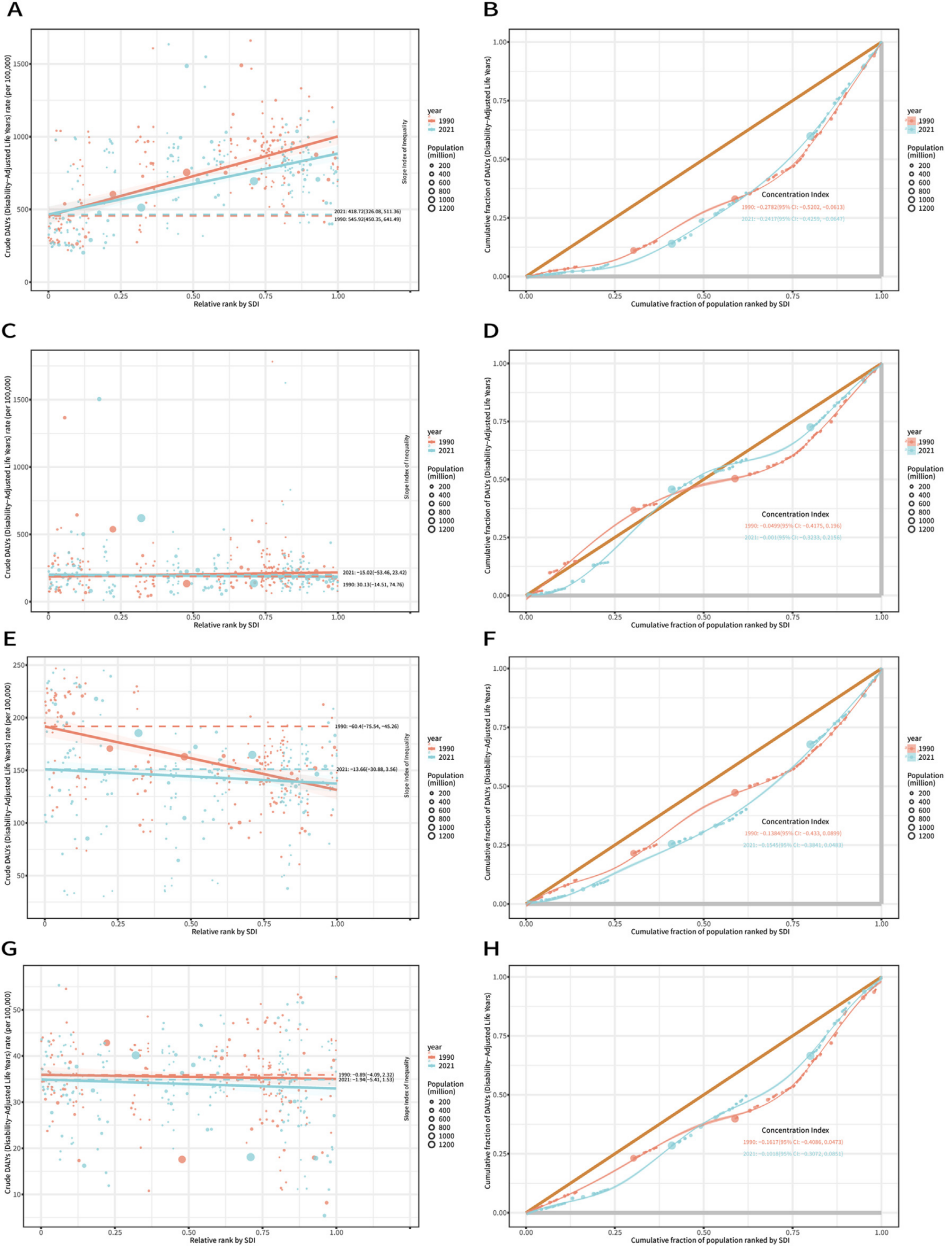
Regression lines of SDI-related health inequalities in the burden of oral disorders in 1990 and 2021. (A) Edentulism, (B) lip and oral cavity cancer, (C) periodontal diseases, and (D) caries of permanent teeth. The concentration curves for SDI-related health inequalities in the burden of oral disorders in 1990 and 2021. (E) Edentulism, (F) lip and oral cavity cancer, (G) periodontal diseases, and (H) caries of permanent teeth. ASDR, age-standardized DALYs rate; DALYs, disability-adjusted life-years; SDI, sociodemographic index.

Through an analysis of inequality in the burden of edentulism, the SII analysis revealed that the disparity in DALY ratios between countries/regions with the highest and lowest SDI decreased from 545.92 in 1990 to 418.72 in 2021. Meanwhile, the CIx value decreased from –0.27 in 1990 to –0.24 in 2021, indicating that although the absolute gap in disease burden between low-SDI and high-SDI countries has narrowed globally, low-SDI countries continue to bear a higher absolute burden. Within individual countries, the disease burden remains disproportionately concentrated in populations with low socioeconomic status, with minimal improvement in the extent of relative inequality (Figure 4A,B).

**Fig. 5:**
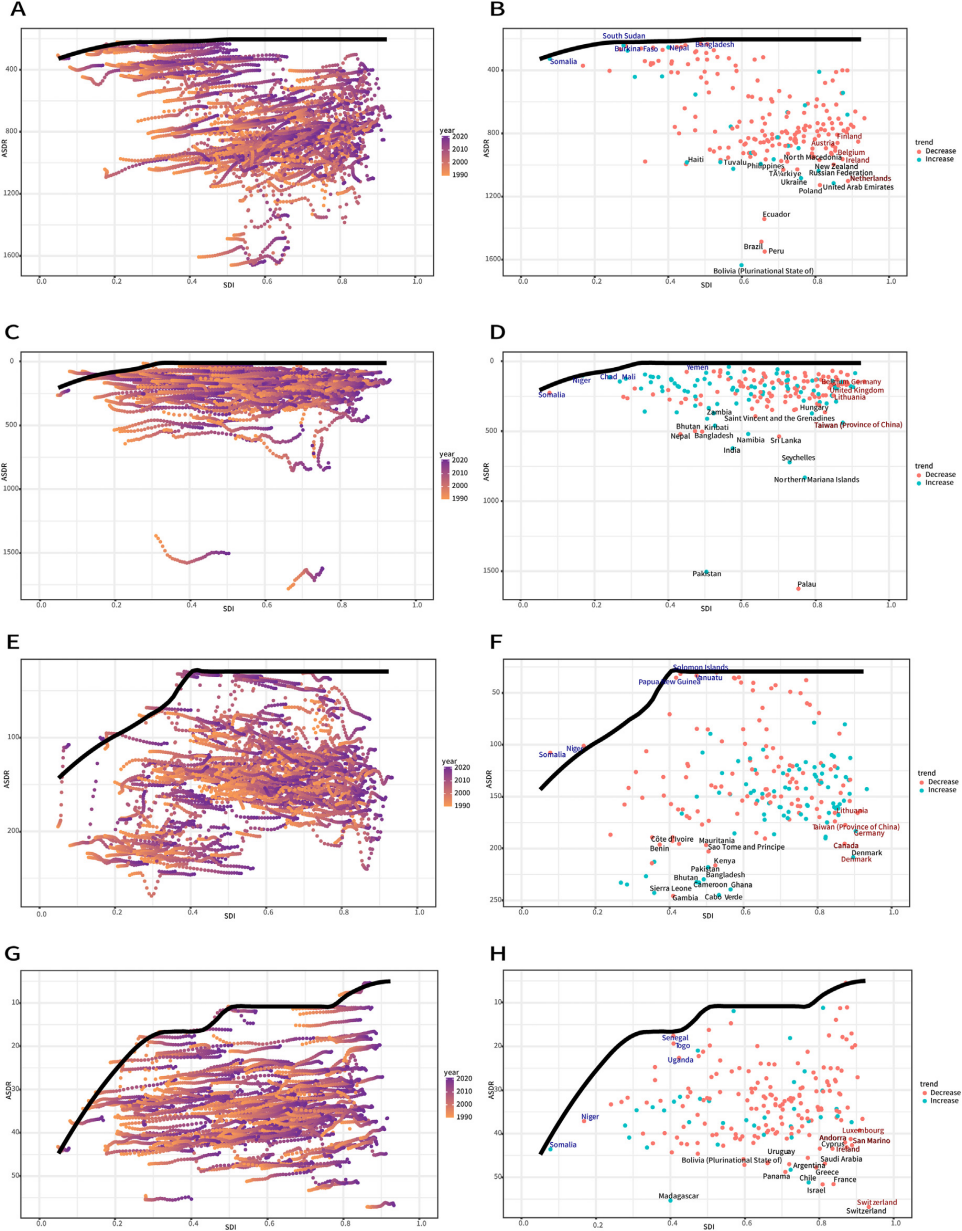
Frontier analyses examining the association between SDI and ASDR for four oral conditions: edentulism (A and B), lip and oral cavity cancer (C and D), periodontal diseases (E and F), and caries of permanent teeth (G and H) across 204 countries and territories. In parts A, C, E, and G, the temporal transition from 1990 to 2021 is depicted by a colour gradient from light orange to dark purple. In parts B, D, F, and H, each point represents a specific country or territory in 2021, with the frontier line shown in black. The top 15 countries and territories with the greatest differences from the frontier are highlighted in brown. Blue colour indicates low-SDI regions with minimal differences from the frontier, while red colour represents high-SDI regions with the greatest differences from the frontier. The direction of ASDR change from 1990 to 2021 is indicated by the colour of dots, with orange dots representing decreases and green dots representing increases. ASDR, age-standardized DALYs rate; SDI, sociodemographic index.

Based on data from 1990 to 2021 and incorporating ASDR and SDI indicators, we performed frontier analysis to assess the potential for improvement of ASDR for edentulism through prevention and treatment across countries and regions with different levels of development (Figure 5A,B). The 15 countries and regions with the largest actual gap in potential improvement include Haiti, Tuvalu, Philippines, Turkey, North Macedonia, New Zealand, Russian Federation, Ukraine, Poland, and the United Arab Emirates. The frontiers with lower SDI values are Somalia, South Sudan, Burkina Faso, Nepal, and Bangladesh. Countries and regions with both high SDI and high development potential, such as Austria, Finland, Belgium, Ireland, and the Netherlands, show relatively high potential for further improvement.

**Fig. 6:**
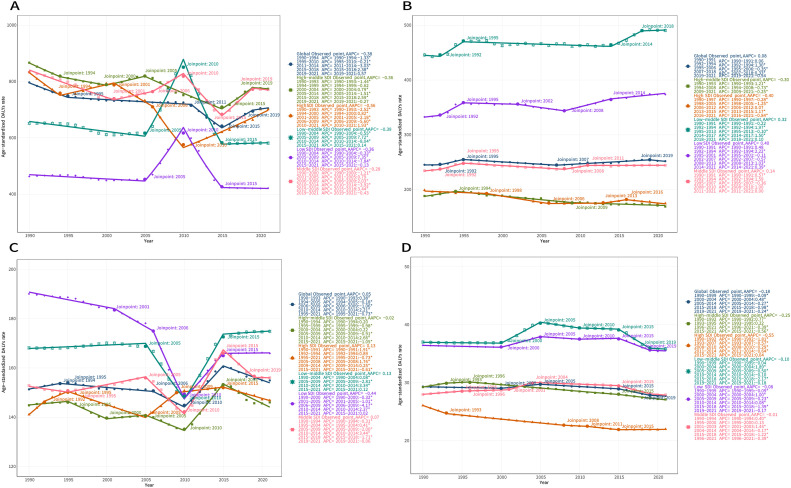
Temporal trends in the age-standardized DALYs rate for (A) edentulism, (B) lip and oral cavity cancer, (C) periodontal diseases, and (D) caries of permanent teeth at the global level and across different SDI regions from 1990 to 2021, based on the Joinpoint regression model. *P < .05; ASDR, age-standardized DALYs rate; DALYs, disability-adjusted life-years; SDI, sociodemographic index.

We analysed the temporal trends of the global burden of DALYs due to edentulism and across different SDI regions using the Joinpoint regression model. The analysis revealed differential changes in the burden of DALYs in each SDI region (Figure 6A). Globally, the burden of DALYs due to edentulism showed a decreasing trend (AAPC = –0.38). Variable decreases were observed during 1990 to 1994 (APC = –1.33), 1995 to 2010 (APC = –0.21), and 2011 to 2014 (APC = –3.03); however, an increasing trend emerged in 2015 to 2018 (APC = 2.38) and 2019 to 2021 (APC = 0.57). Complex fluctuations are found in all SDI regions. The middle SDI region exhibits the most complex trend, characterized by the lowest AAPC value (–0.28) among the SDI regions, as evidenced by a decreasing trend in 1990 to 2000 (APC = –0.28), a decreasing trend in 2001 to 2005 (APC = 0.79), an increasing trend in 2006 to 2009 (APC = 2.03), a pronounced decline in 2010 to 2014 (APC = –3.92), a marked increase in 2015 to 2018 (APC = 3.44), and a decreasing trend in 2019 to 2021 (APC = –0.43).

**Fig. 7:**
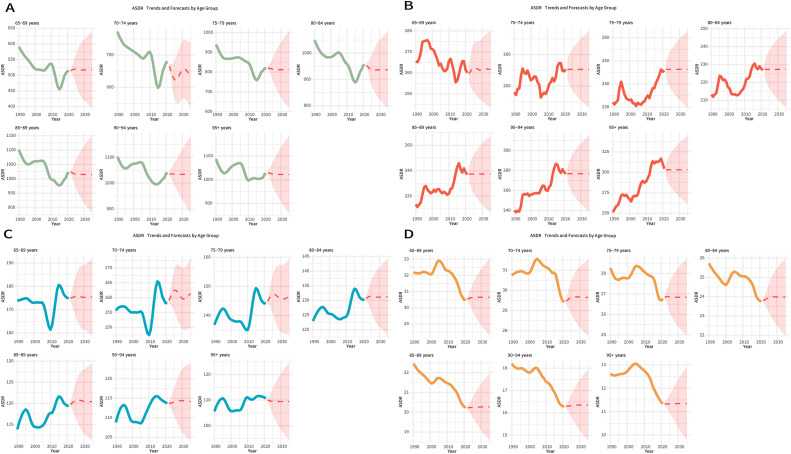
Historical trends and future projections of ASDR for oral disorders across age groups (65-69, 70-74, 75-79, 80-84, 85-89, 90-94, and ≥95 years) from 1990 to 2021 (observed) and from 2022 to 2035 (projected). (A) Edentulism, (B) lip and oral cavity cancer, (C) periodontal diseases, and (D) caries of permanent teeth. ASDR, age-standardized DALYs rate; DALYs, disability-adjusted life years.

When the ARIMA model was applied to predict the disease burden of edentulism from 2021 to 2035, the projected DALY values exhibited considerable variation across different age groups (Figure 7A and Supplementary Table 3). The ASDR for the 65 to 69 years group is projected to continue fluctuating over the next 15 years, reaching 517.47 by 2035. In the 90 to 94 years group, the ASDR is expected to rise to 1033.79 by 2035. The predicted values for other age groups are presented in Table S3.

### Lip and oral cavity cancer

Between 1990 and 2021, the global DALYs for lip and oral cavity cancer increased by 142.0% (from 8.0 to 19.4 million), while the ASDR remained stable (EAPC = 0.03, 95% CI: –0.02 to 0.08). A significant gender reversal was observed: although males continued to bear a higher burden in both years (2021 ASDR: 354.43 vs 164.69 per 100,000 females), the ASDR in males declined significantly (EAPC = –0.19, 95% CI: –0.25 to –0.14), whereas ASDR in females increased (EAPC = 0.26, 95% CI: 0.21-0.31). Socioeconomic disparities widened: low- and low-middle SDI regions exhibited an increasing ASDR (EAPC = 0.26 and 0.20, respectively), in contrast to the significant declines in high-middle SDI regions (EAPC = –0.47, 95% CI: –0.53 to –0.41). Geographically, South Asia carried the highest absolute burden (8.1 million DALYs) in 2021; in contrast, Western Sub-Saharan Africa exhibited the fastest ASDR increase (EAPC = 0.62, 95% CI: 0.57-0.68), while Central Europe also experienced a sharp increase (EAPC = 0.45, 95% CI: 0.34-0.56) (Supplementary Table 4). In the elderly population, the highest number of DALYs was found in the 65 to 69 years group, while the highest ASDR was observed in those aged 95 years and above, with variation in ASDR across different age groups (Figure 3A).

Through an analysis of inequality in the burden of lip and oral cavity cancers, the SII study revealed a striking disparity in DALY ratios between countries/regions with the highest and lowest SDI, which ranged from 30.13 in 1990 to –15.02 in 2021. However, the CIx value showed no significant change. This suggests that, regarding the disease burden of lip and oral cavity cancers, the pattern of global absolute inequality has shifted from a higher burden in high-SDI countries in 1990 to a greater burden in low-SDI countries by 2021. At the same time, within individual countries, the disease burden shifted from being concentrated in low socioeconomic groups to exhibiting minimal socioeconomic disparity (Figure 4C,D).

Based on data from 1990 to 2021 and integrating ASDR and SDI indicators, we used frontier analysis to assess the potential for improvement in ASDR of lip and oral cavity cancer through prevention and treatment across countries and regions with varying development levels (Figure 5C,D). The 15 countries and regions with the greatest actual disparity in potential improvement were Zambia, Saint Vincent and the Grenadines, Hungary, Bhutan, Kiribati, Bangladesh, Nepal, India, Northern Mariana Islands, Pakistan, Seychelles, Sri Lanka, Namibia, Taiwan, and Palau. The frontier countries and regions with lower SDI values included Somalia, Niger, Chad, Mali, and Yemen, while Belgium, Germany, United Kingdom, Lithuania, and Taiwan, all of which have high SDI values and great development potential, showed relatively high potential for improvement.

We examined the temporal trends in the burden of DALYs for lip and oral cavity cancer at the global level and across different SDI regions using Joinpoint regression models. The results revealed differential changes in the burden of DALYs across SDI regions (Figure 6B). Globally, the burden of DALYs in lip and oral cavity cancer showed an increasing trend (AAPC = 0.08). An upward trend with varying rates was observed from 1990 to 1991 (APC = 0.06) to 1992 to 1994 (APC = 1.30), followed by a decreasing trend in 1995 to 2006 (APC = –0.35). Subsequently, the burden increased again from 2007 to 2018 (APC = 0.33), before decreasing from 2019 to 2021 (APC = –0.54). Complex fluctuations were evident across all SDI regions. The trend in the Low-SDI region is more complex (AAPC = –0.28), as indicated by an increase in 1990 to 1991 (APC = 0.48), 1992 to 1994 (APC = 2.22), and 1995 to 2001 (APC = –0.11); this was followed by a decline in 2002 to 2007 (APC = –0.57) and an increase in 2008 to 2013 (APC = 1.03) and 2014 to 2021 (APC = 0.39).

Using the ARIMA model to project the disease burden of lip and oral cavity cancer from 2021 to 2035, the predicted DALY values exhibited considerable variation across different age groups (Figure 7B and Supplementary Table 5). The ASDR of the 80 to 84 years group is projected to continue fluctuating over the next 15 years, reaching 227.28 by 2035, while the ASDR of the 95+ years group is expected to reach 302.99 by 2035. The predicted ASDR values for the remaining age groups are presented in Supplementary Table 5.

### Periodontal diseases

Globally, the DALYs due to periodontal disease increased by 140.0% (4.97-11.94 million), while the ASDR remained stable (EAPC = 0.07, 95% CI: –0.02 to 0.17). Gender differences were minimal, with a slightly higher burden in females (2021 ASR: 149.00/100,000 vs 162.12/100,000 for males). However, the change in ASDR was not significant for either sex. Socioeconomic disparities widened over time, showing significantly decreased ASDR in low-SDI regions (EAPC = –0.66, 95% CI: –0.84 to –0.48). Geographically, Oceania showed the greatest decline in ASDR (EAPC = –5.88, 95% CI: –6.79 to –4.97), whereas Australasia (EAPC = 1.47, 95% CI: 1.09-1.84) and Central Europe (EAPC = 0.53, 95% CI: 0.38-0.67) showed a sharp increase. South Asia carried the highest absolute burden in 2021, with 23.31 million DALYs (Supplementary Table 6). Among older groups, individuals aged 65 to 69 years had the highest number of DALYs and the highest ASDR (Figure 3C).

Through an analysis of inequality in the burden of periodontal disease, the SII study revealed that the disparity in DALY ratios between countries/regions with the highest and lowest SDI levels shifted from –60.4 in 1990 to –13.66 in 2021. However, the CIx value narrowed from –0.14 in 1990 to –0.15 by 2021. This indicates that although low-SDI countries still bear a greater absolute disease burden globally, the absolute gap with high-SDI countries is narrowing significantly. At the national level, the disease burden remains concentrated among populations with lower socioeconomic status, with relative inequality levels showing little change (Figure 4E,F).

Based on data from 1990 to 2021 and incorporating ASDR and SDI indicators, we used frontier analysis to explore the potential for improvement in ASDR for periodontal diseases through prevention and treatment across different countries and regions with varying development levels (Figure 5E,F). The 15 countries and regions with the greatest actual gap in potential improvement were Côte d’Ivoire, Benin, Mauritania, Sao Tome and Principe, Kenya, Gambia, Sierra Leone, Canada, Denmark, Pakistan, Bhutan, Cameroon, Ghana, Cabo Verde, and Bangladesh, while the frontiers with lower SDI values were Somalia, Niger, Papua New Guinea, Vanuatu, and Solomon Islands. Countries and regions with both high SDI and high development potential, such as Lithuania, Taiwan, Germany, Denmark, and Canada, also showed relatively high potential for improvement.

We analysed the temporal trends in the burden of DALYs due to periodontal diseases globally and across different SDI regions using the Joinpoint regression model, which revealed differential changes in the DALY burden within each SDI region (Figure 6C). Globally, the burden of DALYs due to periodontal diseases showed an increasing trend (AAPC = 0.05). From 1990 to 1993, there was an initial rise (APC = 0.38), followed by a decline from 1994 to 2005 (APC = –0.18) to 2006 to 2009 (APC = –0.16). A significant upward trend was then observed from 2010 to 2014 (APC = 2.21). Complex fluctuations were evident across all SDI regions. Among them, the low-SDI region exhibited a more complex pattern (AAPC = –0.44), with decreasing trends during 1990 to 2000 (APC = –0.32), 2001 to 2005 (APC = –1.01), and 2006 to 2009 (APC = –4.17), followed by upward trends in 2010 to 2014 (APC = 2.37) and 2015 to 2021 (APC = 0.02).

When the ARIMA model was applied to predict the disease burden of periodontal diseases from 2021 to 2035, the predicted DALY values showed significant variations across age groups (Figure 7C and Supplementary Table 7). The ASDR of the 95+ years group is expected to continue fluctuating over the next 15 years, with a projected ASDR of 109.46 by 2035. The ASDR of the 65 to 69 years group is projected to reach 175.53 by 2035. The projected ASDR values for other age groups are shown in Supplementary Table 7.

### Caries of permanent teeth

From 1990 to 2021, the global number of DALYs due to permanent dental caries increased by 121.2% (96,617-213,670), while the ASDR decreased significantly (EAPC = –0.12, 95% CI: –0.18 to –0.06). Gender-specific analysis revealed a more pronounced decrease in ASDR among males (EAPC = –0.18, 95% CI: –0.24 to –0.12) compared to females (EAPC = –0.08, 95% CI: –0.14 to –0.01). Socioeconomic inequality increased: regions with high SDI exhibited the greatest improvement (EAPC = –0.52, 95% CI: –0.56 to –0.47), whereas regions with low-middle SDI experienced an increased burden (EAPC = 0.08, 95% CI: –0.07 to 0.23). Geographically, the high-income Asia-Pacific regions exhibited the most rapid decline (EAPC = –0.75, 95% CI: –0.94 to –0.57), whereas tropical Latin America showed the fastest increase (EAPC = 0.18, 95% CI: 0.10-0.25). South Asia bore the highest absolute burden in 2021, with 475,660 DALYs (Supplementary Table 8). Among older age groups, those aged 65 to 69 years had the highest number of DALYs and the highest ASDR (Figure 3D).

Through an analysis of inequality in the burden of caries of permanent teeth, the SII study revealed that the difference in DALY ratios between countries/regions with the highest and lowest SDI values increased from –0.89 in 1990 to –1.94 in 2021; however, the CIx value narrowed from –0.16 in 1990 to –0.1 in 2021. These findings indicate that, at the global level, low-SDI countries have experienced a sustained increase in the absolute disease burden, while the disparity with high-SDI countries has further widened. Within individual countries/regions, although the disease burden continues to be concentrated in low socioeconomic groups, the relative inequality in its distribution has decreased (Figure 4G,H).

Based on data from 1990 to 2021 and incorporating ASDR and SDI indicators, we used frontier analysis to explore the potential for improvement in ASDR of caries of permanent teeth through prevention and treatment across countries and regions with varying development levels (Figure 5G,H). The 15 countries and regions with the greatest actual difference in potential improvement were Madagascar, Bolivia (Plurinational State of), Panama, Uruguay, Argentina, Chile, Israel, Greece, Saudi Arabia, France, Switzerland, Cyprus, Andorra, San Marino, and Ireland. Frontier countries and territories with lower SDI values included Somalia, Niger, Senegal, Uganda, and Togo, contrast with high-SDI regions such as Switzerland, San Marino, Luxembourg, Andorra, and Ireland, which demonstrated high potential for further development.

We analysed the temporal trends in the burden of DALYs due to caries of permanent teeth at the global level and across different SDI regions using the Joinpoint regression model, which revealed differential changes in the DALY burden within each SDI region (Figure 6D). Globally, the burden of DALYs due to caries of permanent teeth exhibited a decreasing trend (AAPC = –0.18). A decreasing trend was observed during 1990 to 1999 (APC = 0.09), followed by a rising trend during 2000 to 2004 (APC = 0.48). However, from 2005 onward, the rates began to decline to varying degrees, especially from 2015 to 2018 (APC = –0.98), where the downward trend was most pronounced. All SDI regions showed a decreasing trend, especially in the high-SDI regions (AAPC = –0.55).

When the ARIMA model was applied to predict the disease burden of caries of permanent teeth from 2021 to 2035, the predicted values of DALYs showed significant variations across age groups (Figure 7D and Supplementary Table 9). Over the next 15 years, the ASDR for the 65 to 69 years group is projected to continue fluctuating, reaching an estimated 30.65 by 2035. Meanwhile, the ASDR for individuals aged 95+ years is expected to reach 11.35 by 2035. The projected values for other age groups are shown in Supplementary Table S9.

### Other oral disorders

From 1990 to 2021, the global number of DALYs due to other oral disorders increased by 132.0% (from 191,413 to 444,114), whereas the ASDR exhibited a slight but statistically significant decline (EAPC = –0.02, 95% CI: –0.03 to –0.02). Gender disparities narrowed slightly over time: although the ASDR remained higher in females than in males (2021: 61.25 vs 53.41/100,000), a significant decline in ASDR was observed only among males (EAPC = –0.02, 95% CI: –0.02 to –0.01). The SDI strata exhibited an opposite trend: high-SDI regions experienced a significant decline (EAPC = –0.07, 95% CI: –0.09 to –0.05), whereas low- and medium-SDI regions showed an increase (EAPC = 0.03, 95% CI: 0.03-0.04 and 0.03, 95% CI: 0.02-0.03). Geographically, central sub-Saharan Africa exhibited the most rapid increase in ASDR (EAPC = 0.04, 95% CI: 0.04-0.05), while high-income North America experienced the steepest decline (EAPC = –0.20, 95% CI: –0.26 to –0.15). Notably, no region recorded an ASDR exceeding 60/100,000 in 2021, suggesting a relatively low but stable global burden (Supplementary Table 10). Among older age groups, those aged 65 to 69 years exhibited the highest number of DALYs and the highest ASDR (Supplementary Figure 2).

Through analysing the inequality in other oral disorders, the SII study found that the disparity in DALY ratios between countries/regions with the highest and lowest SDI decreased from 1.1 in 1990 to 0.37 in 2021. However, the CIx value reflected no significant change, indicating that although high-SDI countries continue to bear a greater absolute disease burdens globally, the absolute gap between low-SDI countries is narrowing. Within individual countries, the disease burden remains concentrated among populations with low socioeconomic status, with relative inequality levels remaining unchanged over decades (Supplementary Figure 3).

Based on data from 1990 to 2021 and incorporating ASDR and SDI indicators, we used frontier analysis to explore the potential improvement in ASDR for other oral disorders through prevention and treatment across different countries and regions with different development levels (Supplementary Figure 4). The 15 countries and regions with the largest actual gap in potential improvement included Russian Federation, Japan, Latvia, Georgia, Taiwan, Lithuania, Sweden, Estonia, Singapore, Norway, Iceland, Monaco, Denmark, Luxembourg, and the United Kingdom, while countries/regions with lower SDI were South Sudan, Afghanistan, Papua New Guinea, Nigeria, and Somalia. Countries and regions with both high SDI and high development potential included Japan, Taiwan, Lithuania, and Sweden.

We analysed the temporal trends in the burden of DALYs for other oral disorders globally and across different SDI regions using Joinpoint regression models, which revealed differential changes in the DALY burden across SDI regions (Supplementary Figure 5). Globally, the burden of DALYs for other oral disorders exhibited a decreasing trend (AAPC = –0.01). Complex fluctuations were observed across SDI regions, with low-middle-SDI and low-SDI regions showing an upward trend.

When the ARIMA model was applied to predict the disease burden of other oral disorders from 2021 to 2035, the projected DALYs exhibited significant variations across different age groups (Supplementary Figure 6 and Supplementary Table 11). The ASDR of the 90 to 94 years group is projected to continue fluctuating over the next 15 years, reaching 27.61 by 2035. The ASDR of the 65 to 69 years group is expected to reach 66.93 by 2035. The projected values for the remaining age groups are shown in Table S11.

## Discussion

This GBD-based study provides a comprehensive, multidimensional assessment of the global burden of oral diseases among older adults. By integrating multiple analytical methods, including Joinpoint regression, health inequality metrics (SII and CIx), frontier analysis, and ARIMA modelling, we aimed to advance the understanding of this public health issue. Our approach enabled a systematic analysis of temporal trends, exploration of socioeconomic inequalities, identification of regions with potential health gains, and projection of future patterns.

Our study reveals a dual character of the global burden of oral diseases among older adults from 1990 to 2021: a consistent increase in the absolute burden alongside divergent trends in the relative burden. On one hand, absolute case numbers increased markedly across all oral diseases (by 112%-142%), with edentulism (+112.2%) and lip and oral cavity cancer (+142.0%) showing the most pronounced growth. On the other hand, the ASDR trend exhibited polarization: declines in ASDR for edentulism (EAPC = –0.47), dental caries of permanent teeth (EAPC = –0.12), and other oral disorders (EAPC = –0.02) reflect progress in prevention and treatment strategies, whereas stagnating ASDR for periodontal diseases (EAPC = 0.07) and lip and oral cavity cancer (EAPC = 0.03) underscores persistent challenges in disease control.

This divergent trend in oral diseases among older adults is closely associated with disease-specific risk factors. The persistently high burden of edentulism (ASDR: 643.21/100,000 for males and 756.53/100,000 for females in 2021) is linked to excessive tooth extractions and inequities in access to prosthodontic care. The higher risk among females may reflect cumulative tooth loss associated with longer life expectancy.^19^ This aligns with recent global analyses indicating that edentulism will continue to pose a significant public health challenge, especially among aging populations, underscoring the need for a sustained public health focus on tooth retention and prosthetic services.^20^ The gender reversal observed in lip and oral cavity cancer (male EAPC = –0.19, female EAPC = 0.26) suggests heightened impacts of tobacco use and human papillomavirus infection among older women.^21^ The global stagnation in the burden of periodontal diseases (EAPC = 0.07) may be attributed to age-related immunosenescence and insufficient primary prevention – only 20% of low-SDI countries have implemented periodontal screening programs.^19^^,^^22^ The paradoxical rise in dental caries in low-middle-SDI regions (EAPC = 0.08) is directly linked to high-sugar diets and insufficient fluoride coverage.^23^ This finding aligns with a recent analysis of six Asian countries, which revealed heterogeneous trends in caries burden, highlighting that although global improvements have been made, they obscure considerable regional disparities and ongoing challenges in specific socioeconomic settings.^24^

This study reveals that the patterns of health inequalities in oral disorders among the global elderly population from 1990 to 2021 are complex and disease-specific. Specifically, for edentulism, the absolute inequality between countries showed significant improvement (with SII decreasing from 545.92 to 418.72), but relative inequality within countries remained persistent (indicated by only a slight change in the CIx from –0.27 to –0.24).^25^ For lip and oral cavity cancer, the disease burden underwent a fundamental shift, transitioning from being concentrated in high-SDI countries to being disproportionately borne by low-SDI countries (as reflected by a change in SII from 30.13 to –15.02), while relative inequality within countries remained stable.^26^ For periodontal diseases, absolute disparities between countries narrowed significantly (the SII decreased from –60.4 to –13.66); however, relative inequality within countries remained largely unchanged (the CIx shifted from –0.14 to –0.15).^27^ In contrast, caries of permanent teeth followed an opposite pattern: absolute inequality between countries widened (the SII increased from –0.89 to –1.94), while relative inequality within countries slightly improved (the CIx increased from –0.16 to –0.10).^28^ These findings underscore the need for future global oral health policies to adopt a dual disease-targeted strategy: not only ensuring the equitable allocation of healthcare resources across countries to mitigate the high burden of lip and oral cavity cancer in low-income countries, but also addressing intracountry health inequalities stemming from socioeconomic disparities, particularly by alleviating the concentrated burden of edentulism and periodontal diseases among disadvantaged populations.^29^

Joinpoint regression analysis revealed several critical transition points in the temporal trajectory of oral disease burden. Edentulism exhibited an adverse upward trend during 2015 to 2018 (APC = 2.38), coinciding with reductions in dental insurance coverage in multiple countries^30^; lip and oral cavity cancer demonstrated sustained growth through 2007 to 2018 (APC = 0.33), paralleling peak global tobacco consumption among females^31^; periodontal diseases showed a marked surge during 2010 to 2014 (APC = 2.21), corresponding to troughs in public health investments^32^; dental caries exhibited accelerated improvement during 2015 to 2018 (APC = –0.98), coinciding with peak fluoridation coverage in high-SDI regions^33^; meanwhile, other oral disorders exhibited no discernible downward inflection points in low-SDI areas, underscoring deficiencies in fundamental diagnostic infrastructure.^34^ Collectively, these temporal inflection points provide valuable historical insights for precision public health interventions.

To address these challenges, frontier analysis across 204 countries and territories reveals divergent improvement potentials for the burden of oral diseases: Somalia, Niger, and other low-SDI regions consistently emerge as frontier performers in theoretical burden reduction, paradoxically underscoring fundamental gaps in primary care services and highlighting the high-impact potential of basic interventions in resource-constrained settings.^35^ Conversely, high-development regions such as Taiwan, Japan, and Lithuania underperform relative to their resource capacity, necessitating enhanced interdisciplinary coordination and longitudinal care mechanisms to complement their advanced screening infrastructure. This is particularly important for maintaining the quality of geriatric oral healthcare despite geographic disparities in economic conditions, nutritional status, and access to healthcare resources.^36^ Together, these findings highlight the need for tailoring oral health policy frameworks to specific regional contexts worldwide.

Looking ahead, ARIMA modelling raises critical warnings for 2035. The ASDR of edentulism among individuals aged 90 to 94 years is projected to reach 1033.79 per 100,000, while the ASDR of lip and oral cavity cancer in those aged ≥95 years is expected to rise to 302.99 per 100,000 – together indicating a surge in complex geriatric treatment needs.^7^^,^^8^ The burden of periodontal diseases is projected to remain high at 175.53 per 100,000 among individuals aged 65 to 69 years, necessitating continued community-based preventive efforts.^37^ The concentration of dental caries in the ‘younger elderly’ cohort (65-69 years, ASDR = 30.65) warrants early restorative interventions.^28^ Meanwhile, the apparent stability of other oral disorders (ASDR <60 per 100,000) may substantially obscure their true burden in rapidly aging populations.^38^

This study has several limitations to be noted. First, subtle variations in multinational epidemiological databases might lead to divergent interpretations when extrapolated to populations with smaller sample sizes. Second, although standardized indices such as the CI and SII are effective for characterizing socioeconomic gradients at the population level, these composite metrics may inadvertently obscure critical contextual determinants – including healthcare policy frameworks and cultural practices – that shape oral disease patterns across national settings. Third, although the GBD study is a robust source of data, its estimates are inherently constrained by the quality and availability of underlying data, particularly in low- and middle-income countries. This may result in underestimation or bias in these settings. Furthermore, as indicated in the Methods section, the category of ‘other oral disorders’ is based on more limited data sources, which calls for caution when interpreting the generalizability and precision of the estimates for this specific category. Fourth, our study period includes the initial phase of the COVID-19 pandemic (2020-2021), a time when routine and elective dental services experienced substantial disruptions worldwide. The GBD 2021 estimates may not fully reflect the immediate and long-term consequences of these interruptions on the incidence, progression, and management of oral disease in the elderly population. The potential impact of the pandemic on the observed trends, especially during 2020 to 2021, should be taken account when interpreting the results. As the GBD study is refined annually with the integration of new data, it enables longitudinal tracking of the evolving associations between socioeconomic trajectories and the burden of oral diseases. Such temporal analyses may provide valuable insights for evaluating the effects of oral health policy implementation among older adults across different stages of development.

## Conclusion

In conclusion, this multidimensional analysis of the global burden of oral disorders among older adults from 1990 to 2021 presents a nuanced epidemiological profile. The significant decline in ASDR for edentulism, dental caries, and other oral disorders reflects encouraging progress in specific domains of prevention and management, likely attributable to improvements in higher-SDI settings. However, this progress remains uneven and is overshadowed by the stagnation in the relative burden of periodontal diseases and lip/oral cavity cancer, the alarming rise in absolute case counts across all oral diseases, and the persistent or widening socioeconomic inequalities. The projected escalation of disease burden among the oldest age groups underscores an impending public health challenge. Our findings advocate for a dual-track strategy: celebrating and consolidating achievements in areas where progress has been made, while implementing targeted, equitable, and resilient interventions to tackle persistent burdens, deep-rooted inequalities, and the complex needs of a rapidly aging global population. Future efforts should prioritize strengthening integrated geriatric oral healthcare and addressing the social determinants of oral health.

## Funding

This study was supported by the Shandong Provincial Natural Science Foundation (No. ZR2025QC908), the National Traditional Chinese Medicine Comprehensive Reform Demonstration Zone Science and Technology Co-construction Project (No. GZY-KJS-SD-2024-106), the Shandong Province Higher Education Institutions ‘Youth Innovation Talent Introduction and Cultivation Plan’ (Public Health Safety Risk Assessment and Response Innovation Team), and the Undergraduate Innovation and Entrepreneurship Training Program of Shandong Second Medical University (No. X2025084).

## Author contributions

Yilin Zhang and Xin Xu contributed equally to data curation, formal analysis, and manuscript drafting. Weilu Pan was responsible for investigation, data validation, and visualization. Cheng Lyu contributed to methodology, software, and data processing. Yuan Tang was involved in conceptualization, project administration, funding acquisition, and critical revision of the manuscript. Jing Li supervised the study, provided resources, reviewed and edited the manuscript, and approved the final version. All authors have read and approved the final manuscript.

## Conflict of interest

The authors declare that they have no known competing financial interests or personal relationships that could have appeared to influence the work reported in this article.

## Appendix Supplementary materials

Supplementary Table 1 DALYs of oral disorders in 1990 and 2021, and the corresponding EAPC from 1990 to 2021. Image, application 1

## Footnotes

Supplementary material associated with this article can be found in the online version at doi:10.1016/j.identj.2025.109345.
