# Supplementary material for: Global Burden of Oral Disorders in Adults Aged ≥65, 1990 to 2021: A Systematic Analysis of Global Burden of Disease Study 2021
Source: Int Dent J. 2025 Dec 24;76(1):109345. doi: 10.1016/j.identj.2025.109345 (PMC12800408; doi:10.1016/j.identj.2025.109345)
Supplement: Supplementary file 1 — Supplementary Table 1 DALYs of oral disorders in 1990 and 2021, and the corresponding EAPC from 1990 to 2021. [file mmc1.pdf]

Supplemental Material

Supplemental Table 1. DALYs of oral disorders in 1990 and 2021, and the corresponding EAPC from 1990 to 2021 1
Supplemental Table 2. Global burden of edentulism in 2021 and its percentage changes from 1990 to 2021. 2
Supplemental Table 3. Historical trends and future projections of ASDR for edentulism across age groups (65–69, 70–74, 75–79, 80–84, 85–89, 90–94, and ≥95 years) from 1990 to 2021 (observed) and 2022 to 2035 (projected). 4
Supplemental Table 4. Global burden of lip and oral cavity cancer in 2021 and its percentage changes from 1990 to 2021. 5
Supplemental Table 5. Historical trends and future projections of ASDR of lip and oral cavity cancer across age groups (65–69, 70–74, 75–79, 80–84, 85–89, 90–94, and ≥95 years) from 1990 to 2021 (observed) and 2022 to 2035 (projected). 7
Supplemental Table 6. Global burden of periodontal diseases in 2021 and its percentage changes from 1990 to 2021. 8
Supplemental Table 7. Historical trends and future projections of ASDR of periodontal diseases across age groups (65–69, 70–74, 75–79, 80–84, 85–89, 90–94, and ≥95 years) from 1990 to 2021 (observed) and 2022 to 2035 (projected). 10
Supplemental Table 8. Global burden of caries of permanent teeth in 2021 and its percentage changes from 1990 to 2021. 11
Supplemental Table 9. Historical trends and future projections of ASDR of caries of permanent teeth across age groups (65–69, 70–74, 75–79, 80–84, 85–89, 90–94, and ≥95 years) from 1990 to 2021 (observed) and 2022 to 2035 (projected). 13
Supplemental Table 10. Global burden of other oral disorders in 2021 and its percentage changes from 1990 to 2021. 14
Supplemental Table 11. Historical trends and future projections of ASDR of other oral disorders across age groups (65–69, 70–74, 75–79, 80–84, 85–89, 90–94, and ≥95 years) from 1990 to 2021 (observed) and 2022 to 2035 (projected). 16
Supplemental Figure 1. Global distribution of ASDR of other oral disorders in 2021. Note: ASDR, age-standardized disability-adjusted life years (DALYs) rates 17
Supplemental Figure 2. The number of DALYs 18
Supplemental Figure 3. SDI-related health inequality regression lines(a) and concentration curves(b) for the burden of Other oral disorders in 1990 and 2021 19
Supplemental Figure 4. Temporal trend changes in Age-standardized DALYs rate for Other oral disorders globally and in various SDI regions from 1990 to 2021 based on the Joinpoint regression model 20
Supplemental Figure 5. Frontier analysis exploring the relationship between SDI and ASDR for Other oral disorders in 204 countries and territories. 21
Supplemental Figure 6. Historical trends and future trajectories of ASDR for Other oral disorders by age group (65–69, 70–74, 75–79, 80–84, 85–89, 90–94, and ≥95 years) from 1990 to 2021 (observed) and 2022 to 2035 (projected) 22

Supplemental Table 1. DALYs of oral disorders in 1990 and 2021, and the corresponding EAPC from 1990 to 2021

year	Cause				
	Periodontal diseases	Edentulism	Lip and oral cavity cancer	Caries of permanent teeth	Other oral disorders
**Cases 1990**	497435.88(189395.85 to 1063760.43）	2558665.88(1620028.74 to 3783410.38）	801472.84(749962.23 to 848670.45）	96616.91(43219.41to182905.66）	191412.53(118301.71 to 287543.03）
**Rate 1990**	151.33 (57.67to323.62）	796.96 (504.97 to 1176.44）	245.36 (229.06 to 260.00）	29.35 (13.11 to 55.62）	57.88 (35.78 to 86.90）
**Cases 2021**	1193527.19 (457812.00 to 2452118.42)	5429543.50 (3500619.32to7755812.35)	1940091.67(1748671.48 to 2091505.46)	213669.52(95534.19 to 406455.09)	444113.99(274008.12 to 666299.93)
**Rate 2021**	154.96 (59.44 to 318.37)	704.94 (454.50 to1006.97)	251.89 (227.04 to 271.55)	27.74 (12.40 to 52.77)	57.66 (35.58 to 86.51)
**EAPC 1990-2021**	0.07 (0.02 to 0.17)	to0.47 (0.58 to 0.36)	0.03 (0.02 to 0.08)	to0.12 (0.18 to 0.06)	to0.02 (0.03 to 0.02)

DALYs,disability-adjusted life-years.EAPC,estimated annual percentage change.

Supplemental Table 2. Global burden of edentulism in 2021 and its percentage changes from 1990 to 2021.

	1990		2021		1990-2021
	DALY casesNo. *102 (95% UI)	Age-standardized DALY rate per 100,000No. (95% UI)	DALY casesNo. *102 (95% UI)	Age-standardized DALY rate per 100,000No.(95% UI)	EAPC of Age-standardized DALY rateNo. (95% CI)
**Overall**	25586.66 [16200.29-37834.1]	796.96 [504.97-1176.44]	54295.43 [35006.19-77558.12]	704.94 [454.5-1006.97]	-0.47 [-0.58 to -0.36]
**Sex**					
Female	15794.61 [10083.93-23238.39]	849.65 [541.8-1249.83]	32259.91 [20871.74-45747.92]	756.53 [489.1-1073.65]	-0.44 [-0.55 to -0.33]
Male	9792.04 [6106.52-14575.87]	727.09 [455.03-1077.82]	22035.52 [14118.74-31708.63]	643.21 [412.51-924.36]	-0.49 [-0.62 to -0.36]
**Socio-demographic index**					
High SDI	8800.71 [5612.22-12964.22]	833.64 [529.8-1229.69]	14769.42 [9483.68-21419.75]	701.22 [448.99-1019.36]	-0.93 [-1.22 to -0.63]
High-middle SDI	7107.58 [4490.16-10496.08]	866.78 [547.45-1277.47]	14239.34 [9097.22-20351.48]	774.4 [494.77-1107.06]	-0.43 [-0.56 to -0.31]
Middle SDI	6186.92 [3884.63-9167.51]	833 [525.28-1227.85]	17326.62 [11132.94-24579.5]	770.58 [495.96-1092.11]	-0.27 [-0.44 to -0.09]
Low-middle SDI	2754.21 [1712.95-4078.46]	651.5 [406.77-960.59]	6421.97 [4132.26-9065.25]	581.26 [375.11-819.22]	-0.12 [-0.54 to 0.31]
Low SDI	699.41 [432.16-1040.99]	463.18 [287.47-686.85]	1476 [949.2-2145.98]	421.39 [271.04-610.72]	-0.08 [-0.5 to 0.35]
**Region**					
Andean Latin America	256.2 [169.03-361.86]	1601.42 [1056.55-2261.33]	758.04 [504.14-1057.2]	1503.56 [998.96-2097.71]	-0.22 [-0.33 to -0.11]
Australasia	292.33 [199.42-402.98]	1305.54 [890.63-1799.75]	494.53 [314.35-721.28]	941.74 [596.33-1376.22]	-0.3 [-0.66 to 0.05]
Caribbean	217.35 [137.71-319.63]	962 [608.75-1413.79]	429.04 [273.98-629.52]	888.37 [566-1305.38]	-0.39 [-0.44 to -0.33]
Central Asia	322.45 [203.82-472.19]	915.14 [577.01-1341.63]	496.84 [310.6-738.83]	845.68 [531.37-1255.5]	-0.48 [-0.67 to -0.29]
Central Europe	1426.22 [915.5-2072.84]	1097.21 [704.06-1592.12]	2188.79 [1395.66-3211.97]	968.4 [616.37-1423.06]	-0.72 [-0.85 to -0.59]
Central Latin America	592.98 [377.12-871.65]	931.51 [592.74-1367.66]	1907.73 [1216-2680.43]	896.01 [570.98-1259.08]	-0.33 [-0.47 to -0.18]
Central Sub-Saharan Africa	58.48 [36.52-85.82]	413.42 [258.73-606.53]	137.41 [85.28-201.62]	415.54 [258.71-610.62]	-0.09 [-0.2 to 0.01]
East Asia	4692.77 [2881.75-7072.08]	748.02 [461.77-1119.41]	13528.65 [8443.86-19795.43]	692.03 [433.14-1009.85]	-0.28 [-0.63 to 0.08]

	1990		2021		1990-2021
	**DALY casesNo. *102 (95% UI)**	**Age-standardized DALY rate per 100,000No. (95% UI)**	**DALY casesNo. *102 (95% UI)**	**Age-standardized DALY rate per 100,000No.(95% UI)**	**EAPC of Age-standardized DALY rateNo. (95% CI)**
Eastern Europe	2237.54 [1419.78-3280.22]	952.48 [602.76-1396.23]	3413.24 [2125.31-4812.65]	1017.18 [632.47-1434]	0.14 [0.07 to 0.22]
Eastern Sub-Saharan Africa	132.72 [80.65-200.41]	268.5 [163.78-404.11]	285.85 [174.59-430.06]	257.57 [157.73-387.03]	-0.18 [-0.23 to -0.13]
High-income Asia Pacific	1174.55 [726.14-1772.3]	674.4 [416.75-1016.8]	2680.35 [1679.87-3942.8]	514.65 [320.69-761.05]	-1.49 [-2.14 to -0.83]
High-income North America	2906.18 [1812.8-4296.54]	834.72 [518.94-1235.63]	4356.99 [2804.71-6315.9]	672.7 [432.44-976.19]	-1.01 [-1.46 to -0.56]
North Africa and Middle East	1190.89 [752.12-1740.82]	982.82 [624.01-1433.08]	3060.67 [1967.86-4392.3]	910.07 [586.76-1303.35]	-0.3 [-0.4 to -0.2]
Oceania	13.89 [8.41-20.91]	780.88 [479.69-1163.84]	33.82 [20.72-50.91]	740.5 [457.21-1106.55]	-0.15 [-0.17 to -0.12]
Southeast Asia	1351.12 [840.09-2016.74]	754.92 [471.07-1123.03]	3330.93 [2129.11-4744.46]	671.1 [430.14-954.99]	-0.37 [-0.41 to -0.34]
South Asia	2107.73 [1276.07-3166.5]	559.99 [341.03-837.28]	5401.82 [3432.69-7690.65]	474.68 [302.43-674.43]	-0.01 [-0.8 to 0.78]
Southern Latin America	376.55 [237.06-556.48]	917.69 [577.32-1355.79]	650.5 [409.72-960.17]	791.72 [497.05-1170.61]	-0.49 [-0.52 to -0.45]
Southern Sub-Saharan Africa	129.44 [81.09-188.43]	623.7 [391.31-907.61]	249.95 [156.99-364.32]	574.75 [361.37-837.27]	-0.49 [-0.8 to -0.17]
Tropical Latin America	1037.85 [688.17-1465.62]	1479.08 [983.71-2083.48]	3285.84 [2147.3-4466.06]	1473.98 [962.85-2003.78]	0.01 [-0.11 to 0.14]
Western Europe	4853.9 [3086.85-7116.77]	855.52 [541.47-1256.56]	7186.23 [4591.09-10477.03]	771.03 [489.66-1128.32]	-0.67 [-1.09 to -0.24]
Western Sub-Saharan Africa	215.52 [132.57-323.27]	337.43 [207.97-504.9]	418.22 [267.82-607.76]	318.66 [204.33-461.93]	-0.54 [-0.7 to -0.37]

DALYs, disability-adjusted life-years.EAPC estimated annual percentage change;SDI, sociodemographic index.

Supplemental Table 3. Historical trends and future projections of ASDR for edentulism across age groups (65–69, 70–74, 75–79, 80–84, 85–89, 90–94, and ≥95 years) from 1990 to 2021 (observed) and 2022 to 2035 (projected).

year	Forecast ASDR（lower_95 to upper_95）						
	age_group 65-69	age_group 70-74	age_group 75-79	age_group 80-84	age_group 85-89	age_group 90-94	age_group 95+
**2022**	514.2589(509.5527to518.965)	665.6389(657.5694to673.7085)	819.192(810.6525to827.7314)	953.4863(944.4272to962.5454)	1024.484(1015.663to9 1033.305)	1036.621(1027.445to7 1045.796)	1031.032(1022.302to21039.762)
**2023**	517.4293(503.2691to531.5894)	649.3994(627.5764to671.2224)	819.6689(799.933to839.4047)	947.2235(927.4675to966.9795)	1019.992(1002.109to5 1037.874)	1036.11(1016.918to6 1055.301)	1027.706(1009.251to61046.161)
**2024**	519.2379(491.4955to546.9803)	634.0556(595.3037to672.8076)	816.4906(779.7409to853.2403)	940.4684(903.8116to977.1251)	1017.664(984.994to0 1050.334)	1035.682(1005.507to8 1065.856)	1025.63(991.112to51060.147)
**2025**	519.4393(474.9938to563.8848)	624.365(568.937to679.793)	814.6195(757.864to871.3751)	936.285(881.5369to991.033)	1016.458(969.703to0 1063.212)	1035.323(993.703to6 1076.943)	1024.333(973.565to01075.102)
**2026**	518.6264(458.2669to578.986)	622.4799(553.3227to691.6372)	813.518(737.5731to889.4629)	934.8454(863.9601to1005.7308)	1015.833(956.45to41075.215)	1035.024(981.802to3 1088.245)	1023.524(957.468to11089.579)
**2027**	517.6027(444.3896to590.8159)	627.6959(548.9599to706.432)	812.8695(719.2719to906.4671)	935.0941(850.9006to1019.2876)	1015.509(944.874to5 1086.143)	1034.773(969.986to9 1099.558)	1023.018(942.864to31103.173)
**2028**	516.9255(434.1638to599.6872)	637.1554(552.6917to721.6192)	812.4878(702.7924to922.1831)	935.9164(840.8804to1030.9524)	1015.341(934.608to0 1096.075)	1034.563(958.371to8 1110.753)	1022.703(929.601to11115.805)
**2029**	516.7563(426.8695to606.6431)	647.1223(559.5874to734.6572)	812.263(687.8599to936.6661)	936.6313(832.4891to1040.7735)	1015.254(925.359to5 1105.149)	1034.387(947.026to8 1121.746)	1022.506(917.488to21127.524)
**2030**	516.9559(421.2805to612.6313)	654.3238(565.0795to743.5682)	812.1307(674.212to950.0495)	937.0102(824.8493to1049.171)	1015.209(916.911to8 1113.507)	1034.239(935.992to9 1132.486)	1022.383(906.346to81138.419)
**2031**	517.2759(416.3196to618.2323)	656.92(566.3852to747.4548)	812.0528(661.6267to962.4789)	937.0993(817.5718to1056.6267)	1015.186(909.105to3 1121.267)	1034.116(925.291to1 1142.941)	1022.306(896.023to21148.589)
**2032**	517.519(411.3279to623.7101)	654.8518(562.801to746.9027)	812.007(649.9249to974.089)	937.0359(810.5552to1063.5165)	1015.174(901.821to8 1128.526)	1034.013(914.929to6 1153.096)	1022.258(886.39to01158.126)
**2033**	517.6069(406.0955to629.1183)	649.562(555.2437to743.8803)	811.98(638.964to984.9959)	936.9374(803.8106to1070.0643)	1015.168(894.972to7 1135.363)	1033.927(904.907to5 1162.946)	1022.228(877.343to41167.113)
**2034**	517.5656(400.7321to634.3991)	643.282(545.6255to740.9384)	811.9641(628.6309to995.2973)	936.8653(797.3612to1076.3694)	1015.164(888.49to51141.838)	1033.854(895.218to0 1172.491)	1022.21(868.799to01175.620)
**2035**	517.469(395.4617to639.4763)	638.1861(536.2437to740.1285)	811.9547(618.8355to1005.074)	936.8333(791.2064to1082.4602)	1015.163(882.323to0 1148.002)	1033.794(885.85to71181.737)	1022.198(860.688to41183.707)

ASDR, age-standardized DALYs rate;DALYs, disability-adjusted life-years

Supplemental Table 4. Global burden of lip and oral cavity cancer in 2021 and its percentage changes from 1990 to 2021.

	1990		2021		1990-2021
	**DALY casesNo. *102 (95% UI)**	**Age-standardized DALY rate per 100,000No. (95% UI)**	**DALY casesNo. *102 (95% UI)**	**Age-standardized DALY rate per 100,000No.(95% UI)**	**EAPC of Age-standardized DALY rateNo. (95% CI)**
**Overall**	8014.73 [7499.62-8486.7]	245.36 [229.06-260]	19400.92 [17486.71-20915.05]	251.89 [227.04-271.55]	0.03 [-0.02 to 0.08]
**Sex**					
Female	2765.58 [2503.58-2985.02]	149.61 [135.18-161.61]	6978.91 [6152.38-7698.56]	164.69 [145.72-181.54]	0.26 [0.21 to 0.31]
Male	5249.15 [4858.24-5660.1]	370.29 [342.23-399.36]	12422.01 [10982.45-13645.2]	354.43 [313.29-389.24]	-0.19 [-0.25 to -0.14]
**Socio-demographic index**					
High SDI	2059.29 [1932.71-2155.85]	198.87 [186.73-208.19]	3628.44 [3253.52-3881.67]	174.73 [158.82-185.99]	-0.42 [-0.52 to -0.32]
High-middle SDI	1563.33 [1467.71-1647.74]	188.37 [176.28-198.78]	3108.1 [2768.53-3426.41]	169.09 [150.61-186.43]	-0.47 [-0.53 to -0.41]
Middle SDI	1810.08 [1663.62-1956.5]	234.31 [214.78-253.55]	5614.08 [4965.57-6257.46]	244.46 [215.95-272.49]	0.04 [-0.01 to 0.1]
Low-middle SDI	2018.14 [1736.47-2308.42]	445.86 [383.05-510.6]	5645.23 [4911.88-6360.91]	490.53 [426.34-553.02]	0.2 [0.13 to 0.27]
Low SDI	554.5 [468.3-643.9]	332.64 [280.84-386.58]	1387.85 [1159.92-1625.36]	373.69 [312.46-437.57]	0.26 [0.18 to 0.34]
**Region**					
Andean Latin America	19.2 [15.86-23.29]	119.9 [99.11-145.46]	55.91 [42.81-71.26]	110.68 [84.74-141.12]	-0.24 [-0.39 to -0.09]
Australasia	48.82 [42.06-56.2]	219.32 [188.68-252.76]	88.99 [74.57-104.54]	167 [140.59-195.95]	-0.89 [-1.18 to -0.6]
Caribbean	69.36 [62.12-77.34]	307.1 [274.82-342.6]	121.38 [101.04-143.59]	254.66 [211.94-301.43]	-0.35 [-0.48 to -0.22]
Central Asia	64.93 [57.91-72.28]	186.9 [166.89-207.93]	105.07 [92-119.95]	173.21 [151.66-197.51]	-0.22 [-0.41 to -0.04]
Central Europe	306.35 [286.21-325.44]	232.68 [216.84-247.59]	596.15 [540.58-649.16]	269.5 [244.63-293.51]	0.45 [0.34 to 0.56]
Central Latin America	104.8 [98.38-110.43]	164.61 [154.2-173.56]	267.98 [234.99-300.99]	126 [110.58-141.49]	-1.03 [-1.14 to -0.92]
Central Sub-Saharan Africa	28.28 [21.17-37.49]	185.17 [136.92-247.21]	67.77 [48.94-90.06]	194.06 [138.4-261.85]	0.14 [0.02 to 0.25]
East Asia	909.52 [774.97-1046.22]	136.09 [116.14-156.22]	2873.04 [2302.08-3532.47]	142.76 [114.53-175.06]	0.3 [0.17 to 0.44]
Eastern Europe	488.85 [459.64-520.59]	210.45 [197.61-224.27]	778.68 [700.78-855.69]	232.89 [209.69-255.94]	-0.03 [-0.2 to 0.14]

	1990		2021		1990-2021
	**DALY casesNo. *102 (95% UI)**	**Age-standardized DALY rate per 100,000No. (95% UI)**	**DALY casesNo. *102 (95% UI)**	**Age-standardized DALY rate per 100,000No.(95% UI)**	**EAPC of Age-standardized DALY rateNo. (95% CI)**
Eastern Sub-Saharan Africa	156.01 [133.22-182.25]	283.43 [241.57-331.02]	327.29 [272.82-384.27]	276.48 [230.82-324.02]	-0.17 [-0.21 to -0.13]
High-income Asia Pacific	198.64 [183.86-209.67]	114.87 [106.03-121.4]	708.31 [590.82-783.48]	136.68 [117.79-149.35]	0.27 [-0.11 to 0.65]
High-income North America	785.7 [733.05-822.19]	229.56 [214.68-240]	1106.39 [1003.5-1180]	170.75 [155.78-181.72]	-0.99 [-1.15 to -0.82]
North Africa and Middle East	101.2 [83.92-118.94]	83.58 [69.02-98.52]	274.8 [234.85-315.56]	82.21 [70.13-94.4]	-0.02 [-0.07 to 0.03]
Oceania	3.05 [2.23-4.01]	155.7 [114.09-203.92]	8.73 [6.41-11.52]	178.35 [131.39-234.91]	0.65 [0.56 to 0.75]
Southeast Asia	538.58 [465.39-621.51]	293.83 [253.54-339.24]	1467.11 [1240.06-1706.61]	292.31 [246.44-340.5]	-0.2 [-0.27 to -0.13]
South Asia	2649.89 [2299.95-2996.55]	641.14 [554.87-726.25]	8064.66 [6976.21-9080.22]	670.5 [579.56-755.86]	-0.06 [-0.18 to 0.05]
Southern Latin America	71.47 [62.25-81.69]	174.17 [151.52-199.17]	113.74 [97.55-130.52]	139.99 [120.26-160.69]	-0.34 [-0.57 to -0.1]
Southern Sub-Saharan Africa	53.75 [38.97-67.13]	253.57 [184.26-316.67]	116.97 [103.61-131.75]	258.68 [228.77-291.58]	-0.08 [-0.31 to 0.14]
Tropical Latin America	178.23 [164.23-191.81]	251.76 [230.9-271.16]	498.14 [447.34-541.26]	223.83 [201.28-243.1]	-0.33 [-0.43 to -0.23]
Western Europe	1178.92 [1092.07-1255.88]	213.75 [198.17-227.85]	1613.98 [1425.02-1753.11]	177.98 [159.62-192.4]	-0.54 [-0.68 to -0.39]
Western Sub-Saharan Africa	59.16 [48.41-69.36]	88.06 [72.04-103.35]	145.84 [121.86-173.7]	106.85 [89.49-126.95]	0.62 [0.57 to 0.68]

DALYs, disability-adjusted life-years.EAPC estimated annual percentage change;SDI, sociodemographic index.

Supplemental Table 5. Historical trends and future projections of ASDR of lip and oral cavity cancer across age groups (65–69, 70–74, 75–79, 80–84, 85–89, 90–94, and ≥95 years) from 1990 to 2021 (observed) and 2022 to 2035 (projected).

year	Forecast ASDR（lower_95 to upper_95）						
	age_group 65-69	age_group 70-74	age_group 75-79	age_group 80-84	age_group 85-89	age_group 90-94	age_group 95+
2022	259.6089(255.7381to263.4797)	255.3047(252.0656to258.5438)	246.3168(243.0588to249.5747)	227.2822(223.8218to230.7426)	237.3444(232.1258to242.563)	276.8455(270.168to283.5229)	302.9885(295.6399to310.337)
2023	260.754(253.5775to267.9304)	255.3047(250.724to259.8855)	246.3168(240.774to251.8595)	227.2822(220.4878to234.0766)	237.3444(227.6134to247.0754)	276.8455(264.5481to289.1429)	302.9885(289.5011to316.4758)
2024	261.8863(251.9772to271.7953)	255.3047(249.6945to260.915)	246.3168(239.1872to253.4463)	227.2822(218.3182to236.2462)	237.3444(224.6105to250.0783)	276.8455(260.7873to292.9036)	302.9885(285.3868to320.5901)
2025	262.5138(250.9863to274.0413)	255.3047(248.8265to261.7829)	246.3168(237.8943to254.7392)	227.2822(216.5798to237.9846)	237.3444(222.1915to252.4973)	276.8455(257.7536to295.9374)	302.9885(282.0666to323.9104)
2026	262.4016(249.9787to274.8245)	255.3047(248.0619to262.5475)	246.3168(236.775to255.8585)	227.2822(215.0867to239.4778)	237.3444(220.1087to254.5801)	276.8455(255.1397to298.5512)	302.9885(279.2054to326.7715)
2027	261.9342(248.9445to274.9238)	255.3047(247.3706to263.2388)	246.3168(235.7738to256.8597)	227.2822(213.7574to240.807)	237.3444(218.2518to256.437)	276.8455(252.8085to300.8825)	302.9885(276.6533to329.3236)
2028	261.4986(247.9799to275.0173)	255.3047(246.7349to263.8745)	246.3168(234.8598to257.7737)	227.2822(212.5476to242.0169)	237.3444(216.5601to258.1287)	276.8455(250.6842to303.0068)	302.9885(274.3275to331.6494)
2029	261.3392(247.1886to275.4898)	255.3047(246.1432to264.4662)	246.3168(234.0135to258.62)	227.2822(211.4298to243.1346)	237.3444(214.9961to259.6927)	276.8455(248.7198to304.9711)	302.9885(272.1768to333.8001)
2030	261.4364(246.5321to276.3407)	255.3047(245.5875to265.0219)	246.3168(233.2218to259.4117)	227.2822(210.3858to244.1786)	237.3444(213.5347to261.1541)	276.8455(246.884to306.807)	302.9885(270.1667to335.8102)
2031	261.6374(245.9403to277.3344)	255.3047(245.0618to265.5476)	246.3168(232.4754to260.1582)	227.2822(209.4027to245.1617)	237.3444(212.1579to262.531)	276.8455(245.1544to308.5366)	302.9885(268.2728to337.7041)
2032	261.7866(245.3487to278.2244)	255.3047(244.5619to266.0475)	246.3168(231.7671to260.8664)	227.2822(208.4709to246.0935)	237.3444(210.8525to263.8363)	276.8455(243.5143to310.1766)	302.9885(266.4771to339.4999)
2033	261.8193(244.7286to278.9099)	255.3047(244.0842to266.5252)	246.3168(231.0918to261.5417)	227.2822(207.5831to246.9813)	237.3444(209.6085to265.0803)	276.8455(241.9513to311.7396)	302.9885(264.7656to341.2114)
2034	261.7616(244.0889to279.4343)	255.3047(243.626to266.9834)	246.3168(230.4451to262.1884)	227.2822(206.7336to247.8308)	237.3444(208.418to266.2708)	276.8455(240.4554to313.2356)	302.9885(263.1275to342.8495)
2035	261.6815(243.4613to279.9017)	255.3047(243.1852to267.4242)	246.3168(229.8239to262.8097)	227.2822(205.9179to248.6465)	237.3444(207.2745to267.4143)	276.8455(239.0185to314.6724)	302.9885(261.5541to344.4228)

ASDR, age-standardized DALYs rate;DALYs, disability-adjusted life-years

Supplemental Table 6. Global burden of periodontal diseases in 2021 and its percentage changes from 1990 to 2021.

	1990		2021		1990-2021
	**DALY casesNo. *102 (95% UI)**	**Age-standardized DALY rate per 100,000No. (95% UI)**	**DALY casesNo. *102 (95% UI)**	**Age-standardized DALY rate per 100,000No.(95% UI)**	**EAPC of Age-standardized DALY rateNo. (95% CI)**
**Overall**	4974.36 [1893.96-10637.6]	151.33 [57.67-323.62]	11935.27 [4578.12-24521.18]	154.96 [59.44-318.37]	0.07 [-0.02 to 0.17]
**Sex**					
Female	2691.61 [1026.9-5752.04]	145.05 [55.34-310.29]	6225.76 [2382.25-12828.23]	149 [57-306.77]	0.08 [-0.02 to 0.17]
Male	2282.75 [868.43-4891.15]	159.56 [60.8-341.29]	5709.52 [2191.29-11694.86]	162.12 [62.24-332.38]	0.06 [-0.04 to 0.16]
**Socio-demographic index**					
High SDI	1455.19 [552.21-3087.17]	141.11 [53.49-299.89]	2956.19 [1120.38-6112.03]	146.89 [55.58-302.7]	0.11 [0.02 to 0.2]
High-middle SDI	1213.17 [460.53-2595.37]	144.86 [55-310.18]	2683.77 [1018.88-5530.85]	146.05 [55.44-300.97]	0.06 [-0.06 to 0.19]
Middle SDI	1218.7 [464.88-2607.77]	152.92 [58.44-326.58]	3633.67 [1412.84-7407.97]	156 [60.68-318.37]	0.15 [0.02 to 0.28]
Low-middle SDI	763.89 [294.45-1630.26]	168.17 [64.9-358.17]	2029.21 [777.88-4086.05]	174.88 [67.11-352.74]	0.02 [-0.16 to 0.19]
Low SDI	318.35 [123.66-672.75]	190.06 [73.82-400.64]	623.06 [236.18-1276.91]	165.95 [63.03-340.78]	-0.66 [-0.84 to -0.48]
**Region**					
Andean Latin America	14.6 [5.61-30.57]	91.04 [34.97-190.64]	44.64 [16.46-94.59]	89.67 [33.04-189.9]	0.27 [0.11 to 0.42]
Australasia	20.85 [7.74-44.78]	93.74 [34.81-201.46]	58.84 [21.28-124.2]	114.48 [41.21-241.28]	1.47 [1.09 to 1.84]
Caribbean	35.29 [13.6-75.11]	156.13 [60.1-332.67]	70.75 [26.41-145.05]	150.05 [55.95-307.2]	-0.01 [-0.11 to 0.09]
Central Asia	50.36 [19.22-107.81]	144.89 [55.31-310.8]	70.44 [25.76-147.62]	114.75 [42.11-240.38]	-0.96 [-1.29 to -0.63]
Central Europe	156.07 [58.91-336.3]	117.79 [44.57-253.98]	285.34 [106.13-593.95]	128.68 [47.79-267.67]	0.53 [0.38 to 0.67]
Central Latin America	98.41 [37.93-209.51]	152.25 [58.72-324.22]	325.09 [124.58-657.66]	153.5 [58.83-310.46]	0.18 [0.11 to 0.24]
Central Sub-Saharan Africa	30.94 [12.16-64.78]	197.29 [77.38-411.66]	50.13 [17.8-105.84]	142.71 [51.13-302.35]	-1.43 [-1.67 to -1.19]
East Asia	1134.11 [432.17-2425.59]	162.98 [62.18-347.73]	3386.23 [1303.21-7021.87]	164.48 [63.38-341.21]	0.17 [-0.04 to 0.37]
Eastern Europe	341.53	146.37 [56.09-312.45]	450.59	134.95 [50.05-277.92]	-0.3 [-0.34 to -0.26]

	1990		2021		1990-2021
	**DALY casesNo. *102 (95% UI)**	**Age-standardized DALY rate per 100,000No. (95% UI)**	**DALY casesNo. *102 (95% UI)**	**Age-standardized DALY rate per 100,000No.(95% UI)**	**EAPC of Age-standardized DALY rateNo. (95% CI)**
	[130.92-727.87]		[167.12-929.24]		
Eastern Sub-Saharan Africa	120.11 [47.03-251.91]	216.91 [84.87-453.79]	209.22 [79.02-423.4]	175.79 [66.53-357.32]	-0.87 [-0.97 to -0.77]
High-income Asia Pacific	237.51 [87.75-516.08]	136.71 [50.48-297.1]	681.39 [249.01-1440.04]	152.44 [55.16-319.73]	0.14 [-0.18 to 0.46]
High-income North America	536.79 [205.12-1140.71]	157.38 [60.02-334.79]	968.98 [371.97-1998.14]	151.75 [58.21-312.63]	-0.28 [-0.45 to -0.11]
North Africa and Middle East	164.3 [61.9-355.06]	130.56 [49.34-281.75]	491.13 [186.78-996.23]	142.01 [54.1-288.57]	0.37 [0.3 to 0.45]
Oceania	3.03 [1.11-6.52]	146.96 [54.54-315.01]	1.92 [0.64-4.42]	39.59 [13.14-91.29]	-5.88 [-6.79 to -4.97]
Southeast Asia	274.67 [103.43-585.6]	145.87 [55.05-310.59]	787.4 [305.08-1608.42]	152.32 [59-311.88]	0.15 [0.08 to 0.22]
South Asia	734.31 [285.95-1565.11]	176.86 [68.89-376.13]	2330.98 [896.22-4666.55]	191.76 [73.82-384.62]	0.08 [-0.18 to 0.35]
Southern Latin America	58.2 [21.95-124.56]	141.88 [53.5-303.79]	119.61 [44.51-247.34]	148.73 [55.26-307.04]	0.2 [0.04 to 0.36]
Southern Sub-Saharan Africa	26.86 [9.91-58.32]	126.33 [46.68-274.28]	45.22 [16.04-97.83]	100.85 [35.93-218.93]	-0.9 [-1.08 to -0.73]
Tropical Latin America	73.79 [28.34-160.68]	101.35 [38.93-220.9]	233.14 [90.56-487.27]	105.21 [40.86-219.84]	0.16 [-0.09 to 0.42]
Western Europe	712.27 [269.72-1508.53]	129.32 [48.87-274.22]	1112.5 [415.92-2335.79]	125.36 [46.61-262.09]	-0.03 [-0.24 to 0.18]
Western Sub-Saharan Africa	150.35 [58.71-314.01]	220.76 [86.19-460.48]	211.74 [79.61-428.32]	153.51 [57.75-311.48]	-1.45 [-1.6 to -1.3]

DALYs, disability-adjusted life-years.EAPC estimated annual percentage change;SDI, sociodemographic index.

Supplemental Table 7. Historical trends and future projections of ASDR of periodontal diseases across age groups (65–69, 70–74, 75–79, 80–84, 85–89, 90–94, and ≥95 years) from 1990 to 2021 (observed) and 2022 to 2035 (projected).

year	Forecast ASDR（lower_95 to upper_95）						
	age_group 65-69	age_group 70-74	age_group 75-79	age_group 80-84	age_group 85-89	age_group 90-94	age_group 95+
**2022**	174.9685(173.9411to175.996)	159.0398(158.0889to159.9908)	144.8899(144.181to145.5987)	130.6746(130.082to131.2671)	119.7462(119.1631to120.3293)	113.6845(113.2697to114.0993)	110.162(109.652to0110.671)
**2023**	175.2035(172.1873to178.2196)	160.326(157.738to162.9141)	145.873(143.6774to148.0685)	131.0797(129.2912to132.8681)	120.1161(118.5824to121.6498)	113.7358(112.5788to114.8928)	109.7705(108.2407to4111.300)
**2024**	175.4825(169.8827to181.0823)	161.5684(156.9591to166.1777)	146.6582(142.4457to150.8706)	131.3595(127.9751to134.7439)	120.458(117.7587to123.1573)	113.8541(111.6908to116.0173)	109.6206(106.5886to8112.652)
**2025**	175.6928(167.4775to183.9081)	162.3584(155.7687to168.9481)	147.2316(140.9158to153.5475)	131.4509(126.3927to136.5091)	120.6977(116.8039to124.5915)	114.0043(110.6866to117.3219)	109.5437(104.8571to9114.230)
**2026**	175.771(165.3714to186.1706)	162.4936(154.3035to170.6836)	147.2699(139.091to155.4489)	131.3897(124.8358to137.9435)	120.81(115.8316to125.7884)	114.1542(109.6526to118.6557)	109.5042(103.3009to3115.707)
**2027**	175.7155(163.7711to187.6599)	162.0174(152.7555to171.2792)	147.0565(137.4739to156.639)	131.2615(123.5024to139.0207)	120.8088(114.9283to126.6893)	114.279(108.6622to119.8958)	109.4839(101.9269to8117.040)
**2028**	175.5737(162.6754to188.4719)	161.1713(151.3163to171.0263)	146.5269(136.0035to157.0502)	131.1486(122.4587to139.8385)	120.7311(114.1432to127.3191)	114.3639(107.7661to120.9617)	109.4735(100.7061to7118.240)
**2029**	175.4148(161.9614to188.8681)	160.2877(150.1525to170.4228)	146.0917(134.9986to157.1848)	131.096(121.6691to140.5228)	120.6207(113.4926to127.7488)	114.404(106.99to121.818)	109.4682(99.6081to9119.328)
**2030**	175.3(161.4868to189.1133)	159.6663(149.3958to169.9369)	145.6896(134.243to157.1362)	131.106(121.0469to141.1651)	120.5149(112.9694to128.0604)	114.403(106.3377to122.4683)	109.4654(98.6081to6120.322)
**2031**	175.2622(161.1328to189.3916)	159.4791(149.1067to169.8515)	145.6147(133.9138to157.3156)	131.1528(120.4983to141.8073)	120.4381(112.5533to128.323)	114.3705(105.7978to122.9433)	109.464(97.6869to1121.241)
**2032**	175.2993(160.7914to189.8072)	159.7299(149.2136to170.2461)	145.6501(133.6998to157.6005)	131.2037(119.9534to142.4541)	120.3996(112.2167to128.5824)	114.3193(105.3517to123.2868)	109.4633(96.8299to1122.096)
**2033**	175.3826(160.3651to190.4)	160.2756(149.4998to171.0515)	145.9327(133.6803to158.1851)	131.2357(119.3806to143.0908)	120.3963(111.9303to128.8623)	114.262(104.9789to123.5451)	109.4629(96.0261to6122.899)
**2034**	175.4722(159.8053to191.1391)	160.8945(149.6949to172.0941)	146.1552(133.5037to158.8067)	131.242(118.7842to143.6997)	120.4176(111.6669to129.1683)	114.2096(104.6602to123.759)	109.4627(95.2672to3123.658)
**2035**	175.5343(159.1384to191.9303)	161.3701(149.609to173.1312)	146.4264(133.2846to159.5683)	131.2291(118.1878to144.2704)	120.4504(111.4057to129.4951)	114.1696(104.3791to123.9601)	109.4626(94.5465to7124.378)

ASDR, age-standardized DALYs rate;DALYs, disability-adjusted life-years

Supplemental Table 8. Global burden of caries of permanent teeth in 2021 and its percentage changes from 1990 to 2021.

	1990		2021		1990-2021
	**DALY casesNo. *102 (95% UI)**	**Age-standardized DALY rate per 100,000No. (95% UI)**	**DALY casesNo. *102 (95% UI)**	**Age-standardized DALY rate per 100,000No.(95% UI)**	**EAPC of Age-standardized DALY rateNo. (95% CI)**
**Overall**	966.17 [432.19-1829.06]	29.35 [13.11-55.62]	2136.7 [955.34-4064.55]	27.74 [12.4-52.77]	-0.12 [-0.18 to -0.06]
**Sex**					
Female	543.06 [242.02-1031.84]	29.17 [12.99-55.48]	1162.11 [519.49-2207.82]	27.86 [12.46-52.93]	-0.08 [-0.14 to -0.01]
Male	423.11 [189.93-799.1]	29.66 [13.28-56.09]	974.59 [436.25-1850.15]	27.63 [12.36-52.45]	-0.18 [-0.24 to -0.12]
**Socio-demographic index**					
High SDI	270.28 [120.75-511.07]	26.08 [11.64-49.35]	437.52 [196.45-831.89]	22.01 [9.88-41.87]	-0.52 [-0.56 to -0.47]
High-middle SDI	245.22 [109.14-466.03]	29.26 [13-55.67]	498.46 [222.28-943.65]	27.13 [12.1-51.35]	-0.33 [-0.37 to -0.28]
Middle SDI	220.86 [98.26-416.65]	27.99 [12.41-52.9]	648.51 [289.56-1232.14]	27.91 [12.45-53.03]	0.06 [-0.04 to 0.15]
Low-middle SDI	167.5 [75.23-318.82]	37.03 [16.6-70.48]	415.38 [188.11-790.25]	35.87 [16.23-68.25]	0.08 [-0.07 to 0.23]
Low SDI	61.01 [27.79-117.74]	36.48 [16.59-70.23]	134.62 [61.14-258.51]	35.57 [16.15-68.18]	0.03 [-0.05 to 0.12]
**Region**					
Andean Latin America	7.67 [3.44-14.34]	47.83 [21.48-89.47]	23.35 [10.55-44.23]	46.7 [21.11-88.42]	-0.07 [-0.08 to -0.06]
Australasia	7.01 [3.07-13.24]	31.34 [13.7-59.3]	14.09 [5.97-27.72]	27.53 [11.67-54.11]	-0.29 [-0.57 to 0]
Caribbean	7.39 [3.2-14.23]	32.7 [14.14-62.97]	13.83 [6.15-26.04]	29.33 [13.05-55.16]	-0.38 [-0.41 to -0.35]
Central Asia	12.09 [5.32-23.46]	34.86 [15.32-67.63]	21.08 [9.27-40.9]	34.36 [15.06-66.77]	-0.12 [-0.17 to -0.07]
Central Europe	41.47 [18.35-78.94]	31.23 [13.76-59.5]	65.76 [28.88-124.47]	29.84 [13.11-56.42]	-0.13 [-0.19 to -0.08]
Central Latin America	20.34 [9.04-38.49]	31.54 [14-59.76]	65.44 [29.07-125.1]	30.92 [13.74-59.08]	0.07 [0.01 to 0.13]
Central Sub-Saharan Africa	5.19 [2.26-10.01]	32.54 [14.12-62.94]	12.06 [5.28-23.48]	33.39 [14.54-65]	0.07 [0.01 to 0.13]
East Asia	120.6 [52.01-234.89]	17.35 [7.44-33.84]	367.77 [160.79-712.08]	17.87 [7.81-34.56]	-0.11 [-0.21 to -0.01]
Eastern Europe	66.22 [28.79-128.09]	28.34 [12.26-54.89]	95.89 [42.02-184.92]	28.75 [12.63-55.5]	0.02 [0.01 to 0.04]

	1990		2021		1990-2021
	**DALY casesNo. *102 (95% UI)**	**Age-standardized DALY rate per 100,000No. (95% UI)**	**DALY casesNo. *102 (95% UI)**	**Age-standardized DALY rate per 100,000No.(95% UI)**	**EAPC of Age-standardized DALY rateNo. (95% CI)**
Eastern Sub-Saharan Africa	19.31 [8.68-37.07]	34.67 [15.56-66.53]	38.8 [17.49-73.6]	31.96 [14.39-60.55]	-0.24 [-0.26 to -0.23]
High-income Asia Pacific	16.89 [7.07-32.71]	9.69 [4.05-18.8]	34.49 [14.67-66.91]	7.9 [3.36-15.24]	-0.75 [-0.94 to -0.57]
High-income North America	61.54 [25.65-121.07]	18.07 [7.53-35.54]	113.42 [48.35-220.95]	17.9 [7.64-34.84]	-0.15 [-0.22 to -0.08]
North Africa and Middle East	49.18 [22.13-93.54]	39.06 [17.53-74.28]	127.37 [57.53-241.5]	36.83 [16.61-69.79]	-0.19 [-0.23 to -0.15]
Oceania	0.84 [0.37-1.59]	41.11 [18.08-78.12]	2.03 [0.88-3.84]	40.48 [17.62-76.66]	-0.03 [-0.06 to 0]
Southeast Asia	68.75 [30.43-130.38]	36.52 [16.12-69.34]	177.62 [78.2-334.95]	34.24 [15.05-64.62]	-0.27 [-0.31 to -0.23]
South Asia	168.4 [76.17-321.92]	40.9 [18.45-78.08]	475.66 [215.79-918.28]	39.26 [17.79-75.72]	0.12 [-0.1 to 0.35]
Southern Latin America	19.58 [8.7-36.56]	47.51 [21.09-88.81]	39.34 [18.22-73.41]	49.01 [22.73-91.47]	0.13 [0.07 to 0.19]
Southern Sub-Saharan Africa	5.36 [2.33-10.4]	24.9 [10.83-48.35]	11.01 [4.77-21.4]	23.94 [10.37-46.51]	0 [-0.05 to 0.05]
Tropical Latin America	25.24 [10.99-47.49]	35.05 [15.24-66.07]	80.81 [35.43-152.22]	36.42 [15.97-68.59]	0.18 [0.1 to 0.25]
Western Europe	226.32 [103.29-428.14]	41.01 [18.72-77.65]	323.81 [148.66-621.69]	37.3 [17.15-71.86]	-0.21 [-0.31 to -0.12]
Western Sub-Saharan Africa	16.82 [7.49-32.51]	24.43 [10.86-47.22]	33.07 [14.77-63.53]	23.63 [10.54-45.36]	-0.05 [-0.08 to -0.03]

DALYs, disability-adjusted life-years.EAPC estimated annual percentage change;SDI, sociodemographic index.

Supplemental Table 9. Historical trends and future projections of ASDR of caries of permanent teeth across age groups (65–69, 70–74, 75–79, 80–84, 85–89, 90–94, and ≥95 years) from 1990 to 2021 (observed) and 2022 to 2035 (projected).

year	Forecast ASDR（lower_95 to upper_95）						
	age_group 65-69	age_group 70-74	age_group 75-79	age_group 80-84	age_group 85-89	age_group 90-94	age_group 95+
2022	30.54026(30.39248to30.68803)	29.46352(29.32342to29.60361)	26.83873(26.75346to26.924)	23.79637(23.69522to23.89753)	20.22992(20.14875to20.31108)	16.28709(16.20389to16.3703)	11.3264(11.267351to11.3854)
2023	30.59041(30.26801to30.91282)	29.5345(29.19013to29.87886)	26.86938(26.62469to27.11406)	23.84355(23.58446to24.10265)	20.23162(20.03206to20.43118)	16.30428(16.11131to16.49724)	11.33094(11.18836to11.4735)
2024	30.61844(30.04297to31.19391)	29.60389(29.02948to30.17829)	26.86894(26.38783to27.35005)	23.89187(23.43934to24.3444)	20.23823(19.89745to20.579)	16.31202(15.97813to16.64591)	11.33463(11.075865to11.5934)
2025	30.6341(29.81498to31.45322)	29.65375(28.85803to30.44947)	26.85687(26.13696to27.57678)	23.93226(23.27441to24.5901)	20.24579(19.75493to20.73665)	16.32902(15.84975to16.80828)	11.33764(10.951908to11.7233)
2026	30.64285(29.60195to31.68375)	29.68012(28.69003to30.67021)	26.84555(25.92058to27.77051)	23.96127(23.10303to24.81952)	20.2526(19.61165to20.89355)	16.33446(15.70663to16.96229)	11.34009(10.823842to11.8563)
2027	30.64774(29.40689to31.88859)	29.68728(28.53459to30.83998)	26.83935(25.74878to27.92991)	23.97908(22.93509to25.02307)	20.25809(19.47203to21.04416)	16.34559(15.57855to17.11263)	11.34208(10.695368to11.9887)
2028	30.65047(29.22874to32.0722)	29.68263(28.39588to30.96937)	26.83775(25.61288to28.06262)	23.98775(22.77676to25.19874)	20.26225(19.33849to21.18601)	16.34695(15.44784to17.24607)	11.3437(10.568582to12.1188)
2029	30.652(29.06537to32.23863)	29.67314(28.27444to31.07184)	26.83858(25.5001to28.17707)	23.98999(22.63102to25.34897)	20.26525(19.21206to21.31845)	16.35313(15.33399to17.37227)	11.34501(10.444695to12.2453)
2030	30.65285(28.91456to32.39115)	29.66372(28.1685to31.15893)	26.83999(25.40032to28.27965)	23.98841(22.49859to25.47824)	20.26735(19.09294to21.44176)	16.35198(15.22132to17.48263)	11.34609(10.324384to12.3677)
2031	30.65333(28.77435to32.5323)	29.65687(28.07514to31.2386)	26.841(25.30758to28.37443)	23.98506(22.37876to25.59137)	20.26878(18.98088to21.55669)	16.3553(15.12333to17.58727)	11.34696(10.207994to12.4859)
2032	30.65359(28.64312to32.66407)	29.6532(27.99113to31.31527)	26.84144(25.21918to28.46371)	23.98136(22.27004to25.69268)	20.26973(18.87535to21.66412)	16.35325(15.02686to17.67963)	11.34767(10.095654to12.5996)
2033	30.65374(28.51952to32.78797)	29.65215(27.91353to31.39076)	26.84147(25.13427to28.54867)	23.97812(22.17066to25.78558)	20.27035(18.77575to21.76495)	16.35523(14.94194to17.76852)	11.34825(9.987354to12.7091)
2034	30.65383(28.40247to32.90518)	29.65273(27.84012to31.46534)	26.84133(25.05276to28.6299)	23.9757(22.07886to25.87255)	20.27075(18.68148to21.86002)	16.35319(14.85796to17.84842)	11.34872(9.883to12.8144)
2035	30.65387(28.29108to33.01666)	29.654(27.76943to31.53857)	26.84119(24.9746to28.70778)	23.97415(21.99305to25.95525)	20.271(18.59196to21.95004)	16.35461(14.78275to17.92647)	11.3491(9.782445to12.9157)

ASDR, age-standardized DALYs rate;DALYs, disability-adjusted life-years

Supplemental Table 10. Global burden of other oral disorders in 2021 and its percentage changes from 1990 to 2021.

	1990		2021		1990-2021
	**DALY casesNo. *102 (95% UI)**	**Age-standardized DALY rate per 100,000No. (95% UI)**	**DALY casesNo. *102 (95% UI)**	**Age-standardized DALY rate per 100,000No.(95% UI)**	**EAPC of Age-standardized DALY rateNo. (95% CI)**
**Overall**	1914.13 [1183.02-2875.43]	57.88 [35.78-86.9]	4441.14 [2740.08-6663]	57.66 [35.58-86.51]	-0.02 [-0.03 to -0.02]
**Sex**					
Female	1139.6 [703.41-1703.79]	61.21 [37.81-91.51]	2546.27 [1558.39-3810.16]	61.25 [37.49-91.7]	-0.01 [-0.01 to 0]
Male	774.53 [474.99-1163.66]	53.49 [32.8-80.22]	1894.87 [1156.12-2846.64]	53.41 [32.6-80.18]	-0.02 [-0.02 to -0.01]
**Socio-demographic index**					
High SDI	596.4 [366.04-889.32]	57.95 [35.61-86.49]	1132.41 [697.06-1684.42]	57.48 [35.39-85.63]	-0.07 [-0.09 to -0.05]
High-middle SDI	492.59 [303.18-742.49]	58.47 [36-88.09]	1067.55 [655.33-1600.37]	58.14 [35.69-87.17]	-0.01 [-0.02 to -0.01]
Middle SDI	465.68 [286.52-703.26]	57.92 [35.64-87.34]	1350.71 [830.91-2034.19]	57.75 [35.53-86.93]	-0.01 [-0.01 to 0]
Low-middle SDI	260.61 [160.67-390.27]	56.8 [35.01-84.94]	668.05 [410.78-997.77]	57.13 [35.13-85.27]	0.03 [0.02 to 0.03]
Low SDI	96.61 [59.42-144.7]	56.64 [34.82-84.7]	218.41 [134.5-325.59]	57.05 [35.13-84.99]	0.03 [0.03 to 0.04]
**Region**					
Andean Latin America	9.28 [5.66-13.89]	57.85 [35.28-86.61]	28.7 [17.51-43.15]	57.65 [35.19-86.71]	-0.01 [-0.01 to 0]
Australasia	12.85 [7.79-19.5]	57.54 [34.93-87.42]	28.98 [17.67-43.31]	57.43 [35.02-85.9]	-0.02 [-0.03 to -0.02]
Caribbean	13.15 [7.97-19.63]	58 [35.14-86.61]	26.92 [16.49-40.38]	57.65 [35.32-86.53]	-0.01 [-0.02 to -0.01]
Central Asia	20.47 [12.41-30.84]	59.1 [35.83-89.11]	35.74 [21.75-53.31]	58.27 [35.46-86.92]	-0.04 [-0.04 to -0.03]
Central Europe	76.81 [47.11-114.58]	57.84 [35.52-86.39]	127.72 [78.49-190.26]	57.93 [35.6-86.31]	0.01 [0 to 0.01]
Central Latin America	37.21 [22.91-55.74]	57.41 [35.37-85.99]	122.09 [74.83-183.13]	57.75 [35.4-86.64]	0.02 [0.01 to 0.02]
Central Sub-Saharan Africa	9.23 [5.6-13.83]	56.89 [34.46-84.96]	21.07 [12.89-31.24]	57.61 [35.22-85.36]	0.04 [0.04 to 0.05]
East Asia	410.84 [252.09-623.58]	58.43 [35.85-88.48]	1201.88 [733.76-1816.74]	58.18 [35.52-87.88]	-0.01 [-0.02 to -0.01]
Eastern Europe	138.89 [85.81-207.84]	59.34 [36.72-88.79]	194.71 [119.32-292.88]	58.55 [35.86-88.1]	-0.03 [-0.03 to -0.02]

	1990		2021		1990-2021
	**DALY casesNo. *102 (95% UI)**	**Age-standardized DALY rate per 100,000No. (95% UI)**	**DALY casesNo. *102 (95% UI)**	**Age-standardized DALY rate per 100,000No.(95% UI)**	**EAPC of Age-standardized DALY rateNo. (95% CI)**
Eastern Sub-Saharan Africa	32.32 [19.96-48.65]	56.99 [35.18-85.6]	70.55 [43.48-105.4]	57.56 [35.47-85.9]	0.04 [0.04 to 0.05]
High-income Asia Pacific	102.07 [62.51-152.34]	58.53 [35.88-87.37]	252.76 [153.3-378.15]	58.42 [35.41-87.65]	-0.01 [-0.01 to 0]
High-income North America	194.55 [119.66-291.12]	57.37 [35.34-85.92]	355.61 [219.38-527.83]	56.37 [34.77-83.71]	-0.2 [-0.26 to -0.15]
North Africa and Middle East	73.08 [44.9-109.52]	57.19 [35.14-85.59]	198.47 [121.87-294.95]	56.81 [34.88-84.37]	-0.02 [-0.02 to -0.01]
Oceania	1.19 [0.72-1.78]	56.87 [34.52-84.81]	2.89 [1.78-4.31]	56.63 [34.86-84.46]	-0.01 [-0.01 to -0.01]
Southeast Asia	110.18 [67.69-165.91]	57.81 [35.49-86.96]	303.28 [186.27-454.44]	57.84 [35.5-86.58]	0.01 [0 to 0.01]
South Asia	235.75 [144.67-354.28]	56.25 [34.52-84.39]	695.67 [429.24-1041.52]	56.81 [35.05-84.97]	0.04 [0.04 to 0.05]
Southern Latin America	23.93 [14.39-35.94]	58 [34.88-87.1]	45.92 [27.61-68.25]	57.68 [34.69-85.78]	-0.01 [-0.02 to -0.01]
Southern Sub-Saharan Africa	12.58 [7.75-18.96]	58.32 [35.89-87.87]	26.74 [16.5-40.06]	57.97 [35.77-86.82]	-0.02 [-0.03 to -0.01]
Tropical Latin America	41.77 [25.52-62.71]	57.4 [35.1-86.12]	127.64 [78.44-190.38]	57.59 [35.39-85.9]	0.01 [0 to 0.01]
Western Europe	318.33 [194.18-474.08]	58.12 [35.5-86.71]	492.82 [299.91-733.78]	57.82 [35.21-86.35]	-0.02 [-0.02 to -0.02]
Western Sub-Saharan Africa	39.66 [24.5-59.4]	57.29 [35.38-85.7]	80.97 [49.58-121.42]	57.58 [35.27-86.29]	0.03 [0.02 to 0.04]

DALYs, disability-adjusted life-years.EAPC estimated annual percentage change;SDI, sociodemographic index.

Supplemental Table 11. Historical trends and future projections of ASDR of other oral disorders across age groups (65–69, 70–74, 75–79, 80–84, 85–89, 90–94, and ≥95 years) from 1990 to 2021 (observed) and 2022 to 2035 (projected).

year	Forecast ASDR（lower_95 to upper_95）						
	age_group 65-69	age_group 70-74	age_group 75-79	age_group 80-84	age_group 85-89	age_group 90-94	age_group 95+
**2022**	67.10598(67.05564to67.15632)	62.13126(62.08126to62.18126)	53.63922(53.59318to53.68526)	43.9539(43.90787to43.99994)	32.9482(32.91303to32.98337)	27.76649(27.72307to27.80992)	28.13374(28.09361to28.17387)
**2023**	67.07321(66.96795to67.17847)	62.06384(61.97448to62.15321)	53.60865(53.53152to53.68578)	43.90891(43.82408to43.99373)	32.92429(32.85175to32.99682)	27.71348(27.62521to27.80176)	28.1016(28.00956to28.19364)
**2024**	67.04581(66.88035to67.21127)	62.00484(61.84913to62.16055)	53.57903(53.46283to53.69522)	43.89894(43.76131to44.03658)	32.90506(32.79242to33.0177)	27.67753(27.52882to27.82623)	28.07799(27.93033to28.22565)
**2025**	67.02289(66.79472to67.25106)	61.96676(61.7309to62.20262)	53.55584(53.40106to53.71062)	43.89894(43.70241to44.09548)	32.8896(32.73598to33.04322)	27.64541(27.43442to27.8564)	28.06626(27.85001to28.28251)
**2026**	67.00372(66.71201to67.29543)	61.94852(61.63238to62.26467)	53.53527(53.34054to53.73)	43.89894(43.65748to44.14041)	32.87717(32.68275to33.07158)	27.62618(27.35063to27.90172)	28.06043(27.77964to28.34122)
**2027**	66.98768(66.63266to67.34271)	61.94381(61.55442to62.33321)	53.51834(53.28411to53.75256)	43.89894(43.61969to44.1782)	32.86717(32.63276to33.10159)	27.61207(27.27486to27.94928)	28.05753(27.71873to28.39634)
**2028**	66.97427(66.55683to67.39172)	61.94604(61.49257to62.39951)	53.50376(53.23034to53.77719)	43.89894(43.58643to44.21146)	32.85914(32.58585to33.13242)	27.60555(27.20972to28.00137)	28.0561(27.66527to28.44692)
**2029**	66.96306(66.4845to67.44161)	61.95044(61.44145to62.45943)	53.49155(53.17973to53.80338)	43.89894(43.55639to44.2415)	32.85267(32.54183to33.16352)	27.6021(27.15224to28.05196)	28.05538(27.61753to28.49323)
**2030**	66.95367(66.41557to67.49178)	61.95443(61.39684to62.51203)	53.48116(53.13177to53.83054)	43.89894(43.52877to44.26911)	32.84748(32.50046to33.19449)	27.60211(27.10244to28.10178)	28.05502(27.5742to28.53585)
**2031**	66.94583(66.34987to67.54179)	61.95707(61.35603to62.55811)	53.47239(53.08643to53.85834)	43.89894(43.50308to44.29481)	32.8433(32.46151to33.22509)	27.60318(27.05798to28.14838)	28.05485(27.53434to28.57536)
**2032**	66.93926(66.28721to67.59132)	61.95838(61.31763to62.59912)	53.46495(53.04343to53.88647)	43.89894(43.47896to44.31893)	32.83994(32.42474to33.25514)	27.60528(27.01825to28.19231)	28.05476(27.49727to28.61225)
**2033**	66.93378(66.22741to67.64014)	61.95875(61.28101to62.63648)	53.45866(53.00262to53.91471)	43.89894(43.45615to44.34174)	32.83724(32.38995to33.28453)	27.60732(26.98175to28.23288)	28.05472(27.4625to28.64693)
**2034**	66.92918(66.17025to67.68812)	61.95863(61.24594to62.67132)	53.45334(52.9638to53.94288)	43.89894(43.43446to44.36343)	32.83507(32.35694to33.31319)	27.60931(26.94797to28.27066)	28.0547(27.42966to28.67973)
**2035**	66.92534(66.11555to67.73514)	61.95834(61.21231to62.70437)	53.44884(52.92681to53.97086)	43.89894(43.41374to44.38415)	32.83332(32.32554to33.34111)	27.61087(26.91608to28.30567)	28.05468(27.39845to28.71092)

DALYs, disability-adjusted life-years.EAPC estimated annual percentage change;SDI, sociodemographic index.

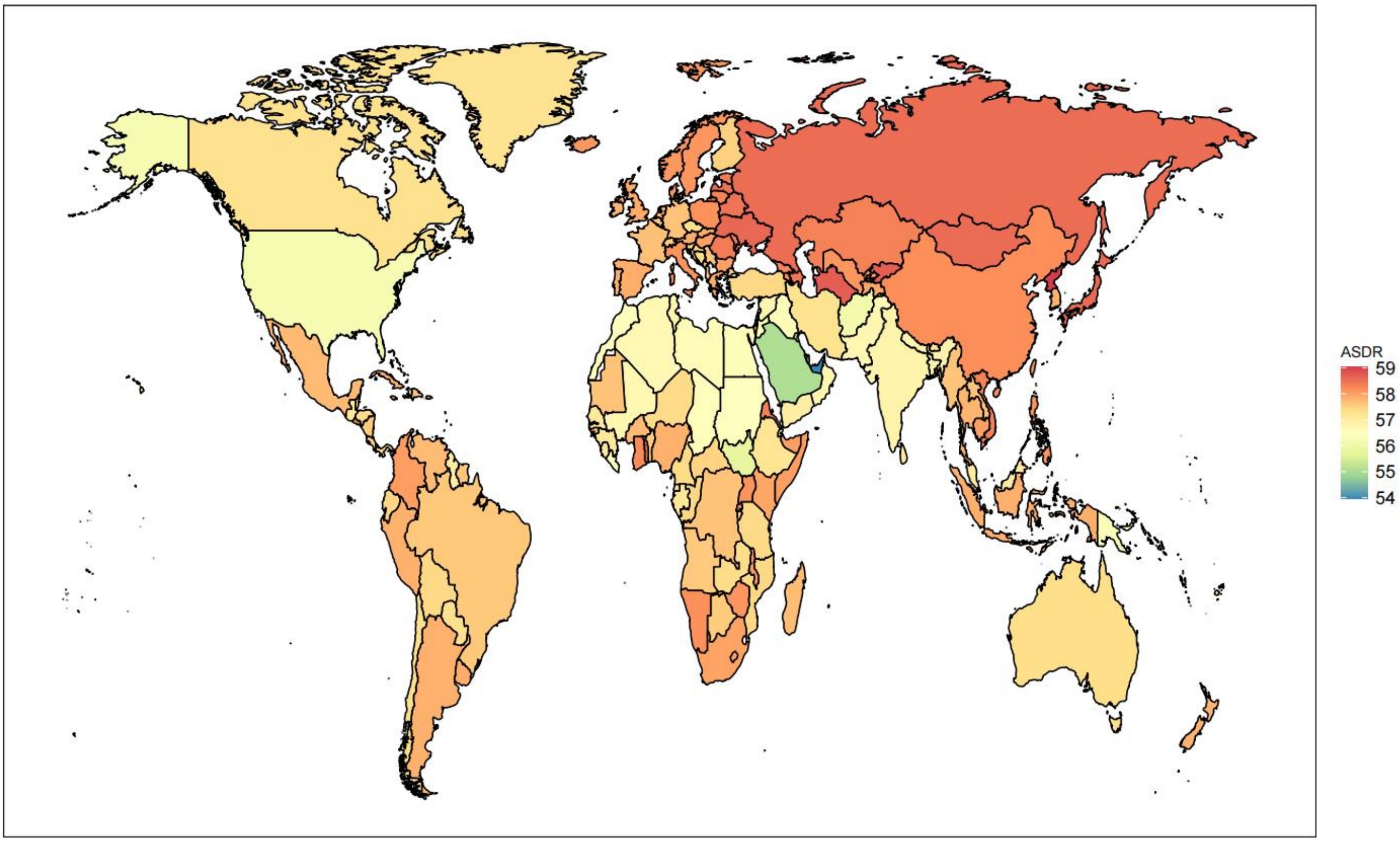

Supplemental Figure 1. Global distribution of ASDR of other oral disorders in 2021. Note: ASDR, age-standardized disability-adjusted life years (DALYs) rates

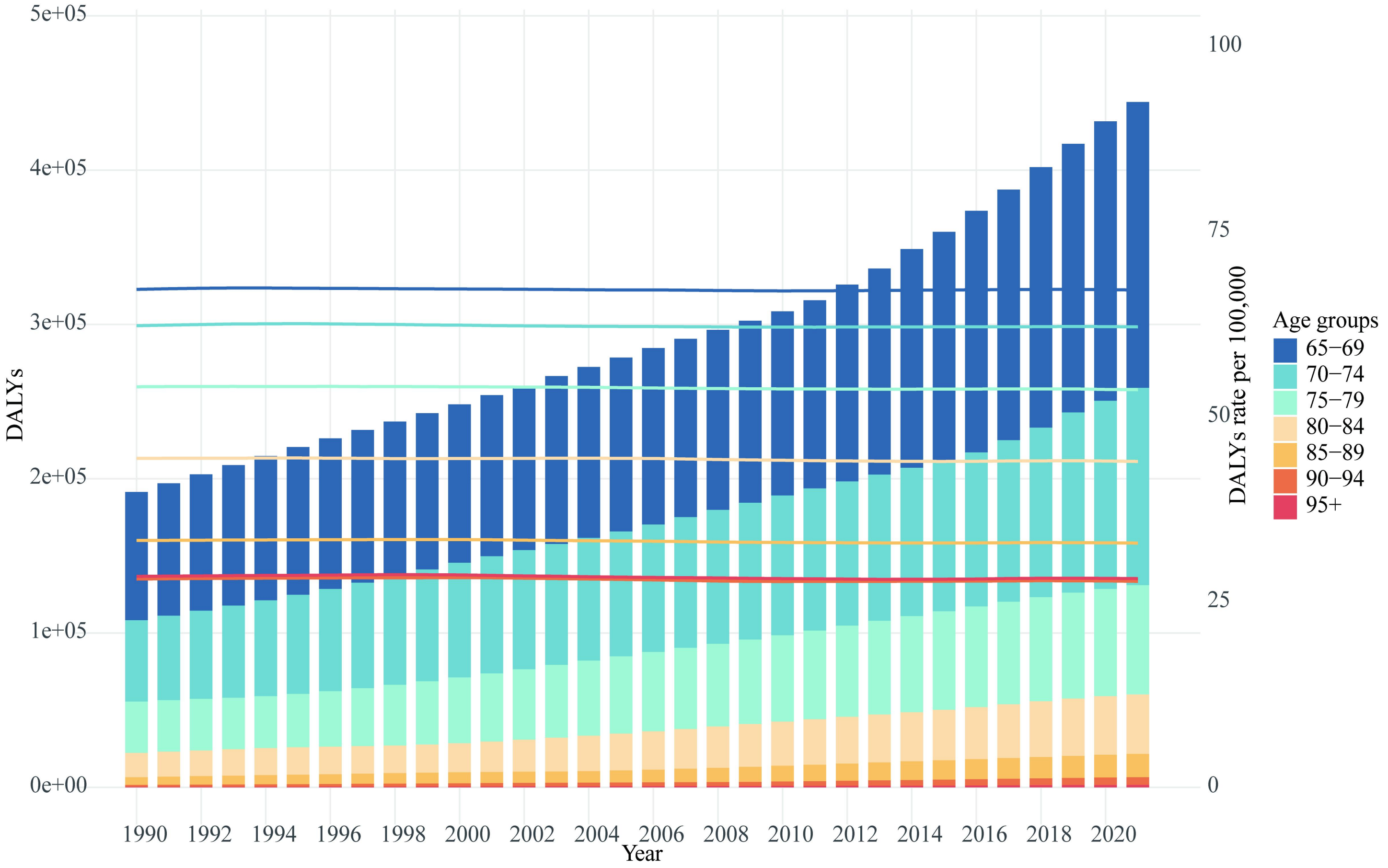

Supplemental Figure 2. The number of DALYs

by age groups (65–69, 70–74, 75–79, 80–84, 85–89, 90–94, and ≥95 years) from 1990 to 2021.

ASDR, age-standardized DALYs rate; DALYs, disability-adjusted life years.

(a) (b)

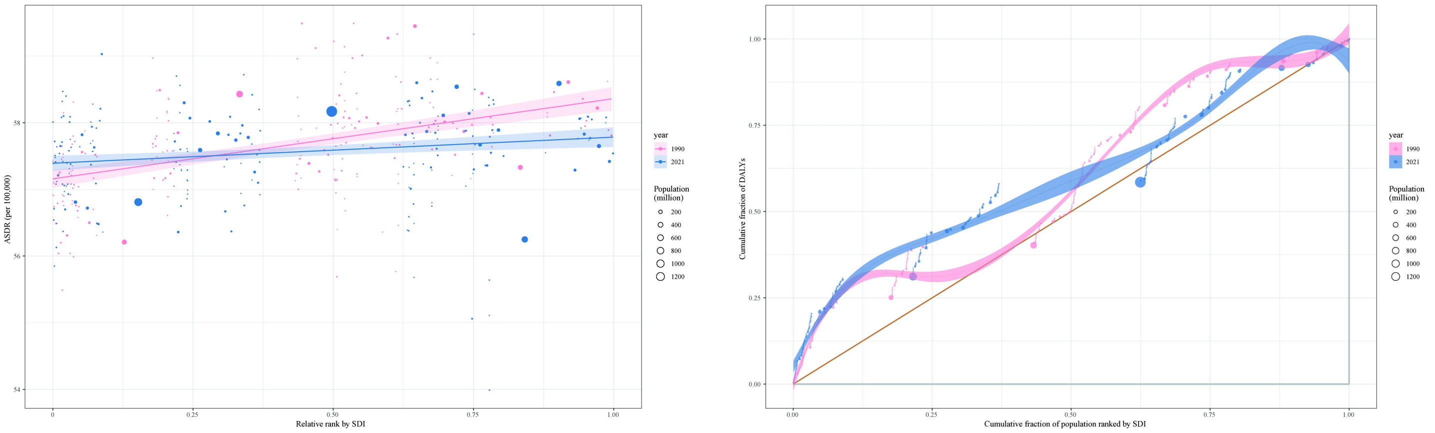

Supplemental Figure 3. SDI-related health inequality regression lines(a) and concentration curves(b) for the burden of Other oral disorders in 1990 and 2021

SDI, sociodemographic index; DALYs , Disability-adjusted life-years;ASDR,Age-standardized DALYs rate.

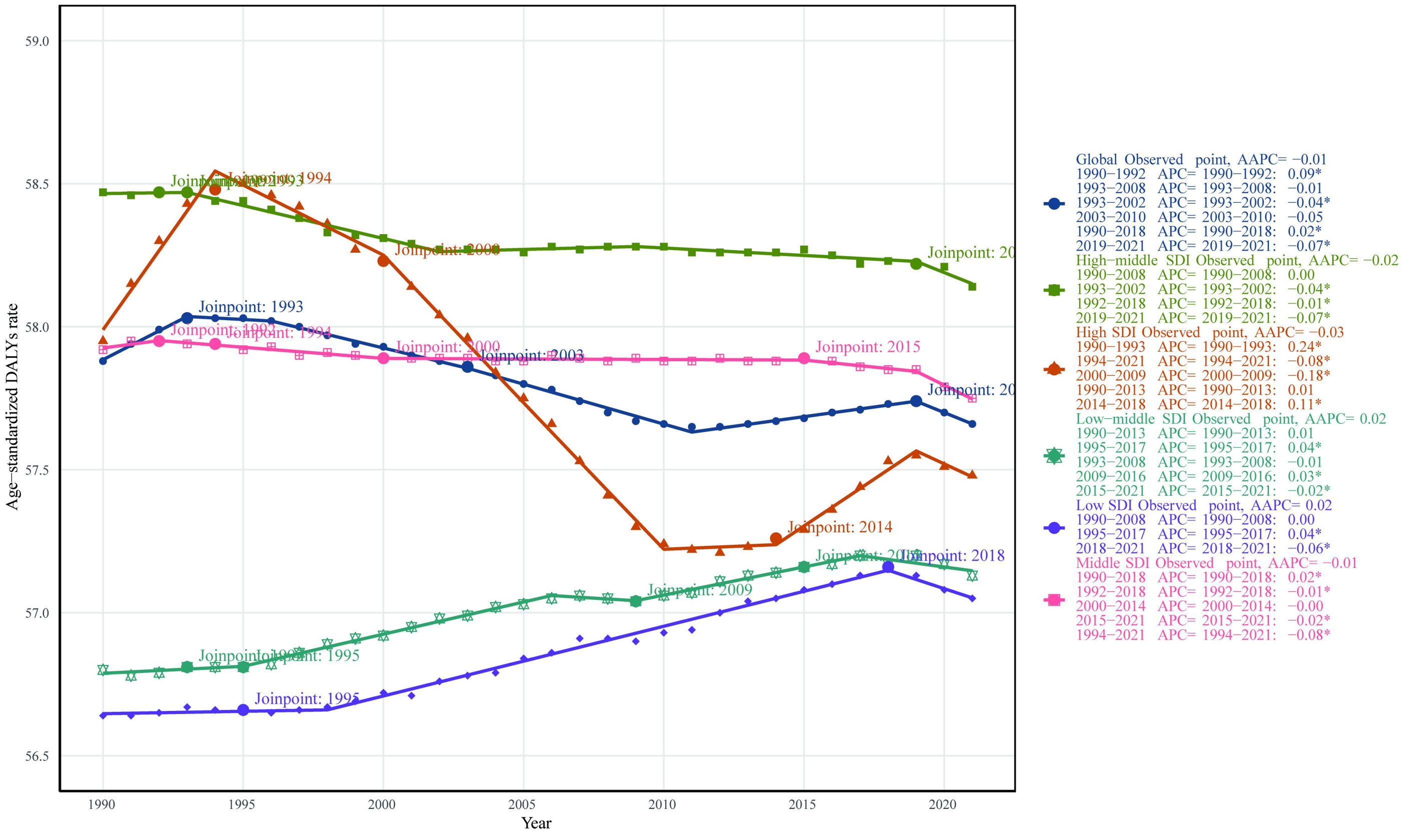

Supplemental Figure 4. Temporal trend changes in Age-standardized DALYs rate for Other oral disorders globally and in various SDI regions from 1990 to 2021 based on the Joinpoint regression model

*$p < 0.05$; SDI, sociodemographic index; DALYs, Disability-adjusted life-years.

(a) (b)

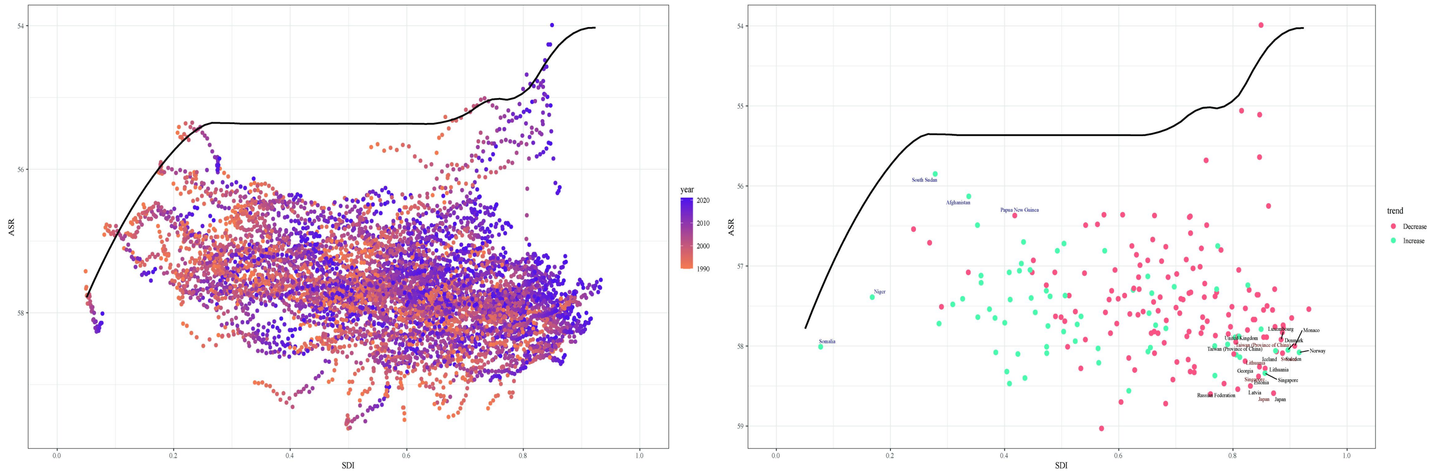

Supplemental Figure 5. Frontier analysis exploring the relationship between SDI and ASDR for Other oral disorders in 204 countries and territories.

In Figures I the color change from light orange (1990) to dark purple (2021) represents the change in years. In Figures J, each point represents a specific country or territory in 2021, the frontier line is shown in black, and the top 15 countries and territories with the largest differences from the frontier are marked in brown. Blue represents low-SDI with the smallest differences from the frontier, red represents high-SDI with the largest differences from the frontier. The direction of ASDR change from 1990 to 2021 is indicated by the color of the dots, with orange dots representing decreases and green dots representing increases. SDI, sociodemographic index; ASDR, Age-standardized DALYs rate.

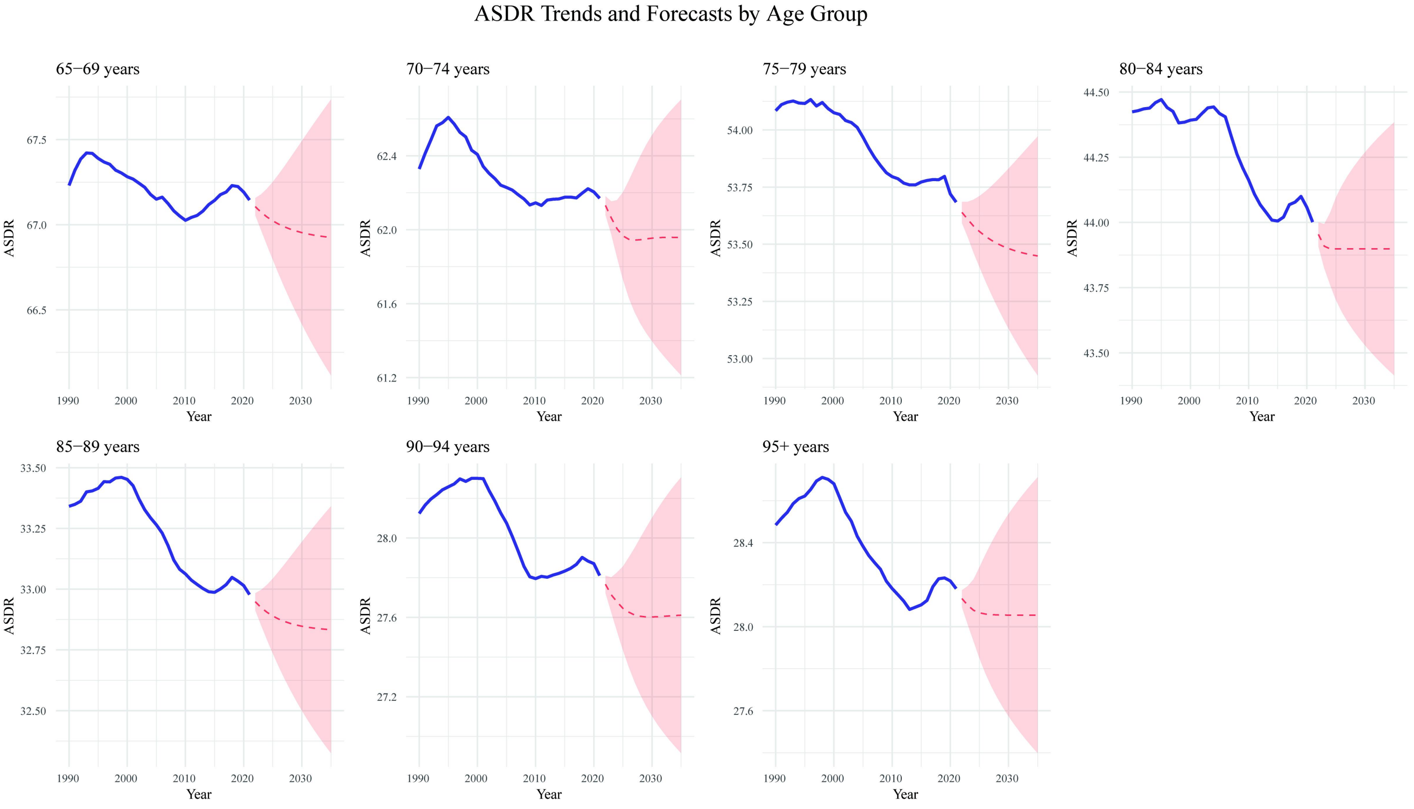

Supplemental Figure 6. Historical trends and future trajectories of ASDR for Other oral disorders by age group (65–69, 70–74, 75–79, 80–84, 85–89, 90–94, and ≥95 years) from 1990 to 2021 (observed) and 2022 to 2035 (projected)

DALYs ,Disability-adjusted life-years;ASDR,Age-standardized DALYs rate.
